# Systemic Lipid Peroxidation and Colorectal Cancer Risk: A Time‐Varying Relationship

**DOI:** 10.1002/ijc.70585

**Published:** 2026-06-15

**Authors:** Gong Yang, Ginger L. Milne, Marina S. Nogueira, Yu‐Tang Gao, Qing Lan, Haoyang Yi, Xiao‐Ou Shu, Wei Zheng, Qingxia Chen

**Affiliations:** ^1^ Division of Epidemiology, Department of Medicine Vanderbilt‐Ingram Cancer Center, Vanderbilt Epidemiology Center, Vanderbilt University Medical Center Nashville Tennessee USA; ^2^ Division of Clinical Pharmacology, Department of Medicine Vanderbilt University Medical Center Nashville Tennessee USA; ^3^ Shanghai Cancer Institute, Renji Hospital, Shanghai Jiaotong University School of Medicine Shanghai China; ^4^ Division of Cancer Epidemiology and Genetics Occupational and Environmental Epidemiology Branch, National Cancer Institute Bethesda Maryland USA; ^5^ Department of Biostatistics Vanderbilt University Medical Center Nashville Tennessee USA

**Keywords:** colorectal cancer risk, lipid peroxidation, nested case–control study, oxidative stress, time‐varying association

## Abstract

Despite widespread interest, large randomized controlled trials have failed to demonstrate chemopreventive benefits of antioxidant supplementation, raising concerns about its efficacy and safety. Building on our prior observation of a time‐dependent inverse association between systemic oxidative stress (OxS), assessed using nucleic acid oxidation biomarkers, and colorectal cancer (CRC) risk, we extended this investigation to systemic lipid peroxidation and evaluated whether a composite OxS index incorporating DNA, RNA, and lipid markers improves risk characterization. We conducted a nested case–control study within two Shanghai cohorts (1938 CRC cases) and replicated findings in a US cohort (285 cases). Systemic lipid peroxidation was assessed using urinary F_2_‐isoprostanes (F_2_‐IsoPs), quantified by UPLC‐MS/MS. Conditional logistic regression estimated odds ratios (ORs) for CRC risk. Lower levels of 5‐F_2t_‐IsoP, a major F_2_‐IsoP isomer generated exclusively via free radical oxidation, were associated with increased CRC risk in both the primary and replication cohorts. Time‐dependent associations were evaluated in the Shanghai cohorts. For CRC diagnosed within 5 years following enrollment, multivariable‐adjusted ORs (95% CI) at the 10th and 90th percentiles of 5‐F_2t_‐IsoP levels, relative to the median, were 1.57 (1.26–1.96) and 0.61 (0.42–0.89), respectively, indicating a 2.2‐fold difference in risk. The composite OxS index showed an even stronger association (3.9‐fold difference). No significant associations were observed for diagnoses beyond 5 years. This study provides new evidence that systemic OxS is inversely and time‐dependently associated with CRC risk during later stages of disease development, raising concerns that lowering systemic OxS via high‐dose antioxidant supplementation potentially carry unintended risks for high‐risk individuals.

## Abstract (highlights)

What's new?

Experimental studies in animals suggest that oxidative stress (OxS) declines as cancer progresses. Whether this occurs in humans, however, remains unclear. The authors of the present study addressed this question by analyzing lipid, DNA, and RNA oxidation biomarkers in relation to colorectal cancer (CRC) risk in patients from Shanghai and the United States. Lower levels of systemic lipid peroxidation were associated with higher CRC risk, particularly within five years of diagnosis. A composite OxS index showed even stronger associations. The findings suggest that declining systemic OxS accompanies later stages of cancer development, with high‐dose antioxidant supplementation potentially worsening cancer risks.

## Graphical abstract

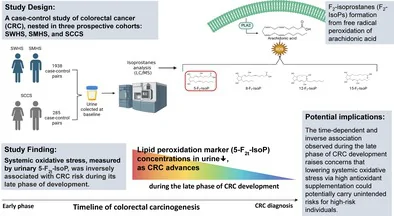

**Abbreviations**
15‐F_2t_‐IsoP (also known as 8‐iso‐PGF_2a_): 15‐F_2t_‐Isoprostane
15‐F_2t_‐IsoP‐M: 2,3‐dinor‐5,6‐dihydro‐15‐F_2t_‐Isoprostane
5‐F_2t_‐IsoP (also known as 5‐iPF_2α_‐VI): 5‐F_2t_‐Isoprostane
8‐oxo‐dG: 8‐oxo‐7,8‐dihydro‐2′‐deoxyguanosine
8‐oxo‐Guo: 7,8‐dihydro‐8‐oxo‐guanosine
CI: confidence interval
CRC: colorectal cancer
F_2_‐IsoPs: F_2_‐isoprostanes
GC‐NICI‐MS: gas chromatography‐negative ion chemical ionization‐mass spectrometry
ng/mg cr: nanograms per milligram of creatinine
OR: odds ratio
OxS: oxidative stress
RCT: randomized controlled trials
RPS‐IsoP: the ratio of 15‐F_2t_‐IsoP‐M (metabolic product) to 15‐F_2t_‐IsoP (substrate)
SCCS: Southern Community Cohort Study
SMHS: Shanghai Men's Health Study
SWHS: Shanghai Women's Health Study
UPLC‐MS/MS: ultra‐performance liquid chromatography tandem mass spectrometry

## 1 Introduction

We recently reported that systemic oxidative stress (OxS), assessed using urinary biomarkers of DNA (8‐oxo‐dG) and RNA (8‐oxo‐Guo) oxidation, was inversely associated with colorectal cancer (CRC) risk in a nested case–control study within three large prospective cohorts: the Shanghai Women's Health Study (SWHS), the Shanghai Men's Health Study (SMHS), and the Southern Community Cohort Study (SCCS) [1].

The inverse association was most evident among cases with shorter lag times between urine collection and CRC diagnosis. These findings provide the first population‐based evidence that a time‐dependent relationship between OxS and tumorigenesis, previously characterized at the cellular level in experimental models [2, 3, 4], may also be reflected systemically in humans. Moreover, this observation may offer a potential mechanistic perspective on a longstanding paradox in cancer prevention: why antioxidant supplementation, intended to reduce systemic OxS, has led to detrimental outcomes among high‐risk individuals in large randomized controlled trials (RCTs) [5, 6, 7].

Importantly, nucleic acid oxidation biomarkers reflect both free radical‐induced formation and enzymatic repair activity of oxidative damage [8]. Thus, interindividual variability in DNA repair capacity may confound the OxS assessment, complicating causal inference and limiting the ability to attribute observed associations solely to free radical‐induced damage. Furthermore, oxidative damage to different macromolecules, including membrane lipids, nuclear DNA, and cytoplasmic RNA, may exert diverse and potentially synergistic effects on tumorigenesis [9].

In this study, we sought to replicate and extend our prior time‐stratified findings by examining lipid peroxidation biomarkers in relation to CRC risk using the same nested case–control design and time‐varying analytic approach in the same source populations. We also evaluated whether a composite OxS index integrating DNA, RNA, and lipid markers improves risk characterization.

## 2 Methods

### 2.1 Study Population

This study used data from the SWHS and SMHS, two large population‐based prospective cohorts in Shanghai, China, for the primary analysis and from the SCCS, a US population‐based prospective cohort for replication. Details of the SWHS and SMHS designs have been previously described [10, 11]. Briefly, the SWHS enrolled 74,941 women aged 40–70 years between 1996 and 2000, while the SMHS enrolled 61,480 men aged 40–74 years between 2002 and 2006. The SCCS, conducted across 12 southern US states, enrolled over 85,000 participants aged 40–79 years between 2002 and 2009 [12]. Baseline data were collected through in‐person interviews, and spot urine samples were obtained from 87.7% of SWHS, 89.1% of SMHS, and 27.6% of SCCS participants.

### 2.2 Follow‐Up and Cancer Ascertainment

Details on cohort follow‐up were described in Supporting Information Methods and Figure S1. Briefly, participants in the SWHS and SMHS were followed for cancer incidence through periodic home visits and annual linkage to the Shanghai Tumor Registry and Shanghai Vital Statistics. Diagnoses were confirmed via medical record review. In the SCCS, incident cancer cases were identified through linkage with 12 state cancer registries and the National Death Index. Only participants who were cancer‐free at baseline and developed CRC as their first primary malignancy during follow‐up were included.

### 2.3 Nested Case–Control Study

A nested case–control design was employed. In the SWHS and SMHS, 1938 incident CRC cases (1035 women and 903 men) with baseline urine samples were identified over a median follow‐up of 15.1 years. Each case was individually matched to one control using incidence‐density sampling based on age at baseline (±2 years), sex, date (±30 days), and time (morning/afternoon) of urine collection, and time interval since the last meal (±2 h). In the SCCS, 294 incident CRC cases were identified among cancer‐free participants with baseline urine samples. Nine cases (3.1%) who were neither Black nor White were excluded due to limited statistical power, leaving 285 cases for this analysis. Each case was matched to two controls using the same criteria as in the Shanghai cohorts, with the addition of race.

### 2.4 Laboratory Measurement

Three urinary biomarkers of lipid peroxidation were evaluated: 5‐F_2t_‐IsoP (also known as 5‐iPF_2α_‐VI), 15‐F_2t_‐IsoP (also known as 8‐iso‐PGF_2a_), and 2,3‐dinor‐5,6‐dihydro‐15‐F_2t_‐IsoP (15‐F_2t_‐IsoP‐M, a major β‐oxidation metabolite of 15‐F_2t_‐IsoP). These were selected based on their abundance and biological relevance [13, 14]. Notably, 5‐F_2t_‐IsoP is generated exclusively via free radical‐induced oxidation [15], while 15‐F_2t_‐IsoP can also be produced enzymatically via cyclooxygenase (COX) pathways [16, 17, 18].

Urinary biomarkers were quantified using ultra‐performance liquid chromatography tandem mass spectrometry (UPLC‐MS/MS) at the Vanderbilt Eicosanoid Core Laboratory (RRID:SCR_010177) (Supporting Information Methods). Case–control pairs were analyzed in the same intra‐day batch. Assay coefficients of variation (CVs) were typically < 8%. Biomarker concentrations were normalized to urinary creatinine and expressed as nanograms per milligram of creatinine (ng/mg Cr).

### 2.5 Statistical Analysis

Primary analyses were conducted in the SWHS and SMHS, with replication in the SCCS. Conditional logistic regression models were used to estimate odds ratios (ORs) and 95% confidence intervals (CIs), stratified by matched case–control sets. The model adequacy for conditional logistic regression was assessed using deviance residual diagnostics (Supporting Information Methods; Table S1). Restricted cubic spline functions with three knots were applied to assess potential nonlinear associations [19], and the exposure and outcome relationship was graphically illustrated. ORs were calculated for the 10th, 30th, 70th, and 90th percentiles of biomarker levels, using the 50th percentile as the reference. Covariates included in the multivariable models are listed in Table 1. To explore potential time‐varying relationships, CRC cases were stratified by time from enrollment to diagnosis into three non‐overlapping intervals: < 5 years, 5–9 years, and > 9 years; cut points were chosen to reflect the distribution of follow‐up time to diagnosis (Figure S2). Time‐stratified analyses were not feasible in SCCS due to limited case numbers. SCCS was used to replicate overall associations.

**TABLE 1:** Associations between baseline urinary levels of F_2_‐IsoPs and subsequent risk of colorectal cancer.

	Number of pairs^c^	OR (95% CI) for colorectal cancer risk relative to reference,^a^ by percentile distribution of biomarkers^b^					*p* for overall association	*p* for nonlinear association
		10th	30th	50th	70th	90th		
In the SWHS and the SMHS								
5‐F_2t_‐IsoP	1778							
Age adjusted		1.13 (1.05, 1.22)	1.06 (1.03, 1.09)	Reference	0.93 (0.89, 0.97)	0.82 (0.71, 0.96)	< 0.001	0.156
Multivariable adjusted^d^		1.12 (1.04, 1.21)	1.05 (1.02, 1.08)	Reference	0.94 (0.90, 0.97)	0.84 (0.73, 0.97)	0.001	0.177
15‐F_2t_‐IsoP	1617							
Age adjusted		1.00 (0.95, 1.05)	1.00 (0.99, 1.02)	Reference	0.99 (0.96, 1.01)	0.94 (0.87, 1.01)	0.255	0.543
Multivariable adjusted^d^		0.99 (0.94, 1.04)	1.00 (0.98, 1.01)	Reference	0.99 (0.96, 1.02)	0.94 (0.87, 1.02)	0.263	0.368
15‐F_2t_‐IsoP‐M	1919							
Age adjusted		1.11 (1.05, 1.17)	1.05 (1.02, 1.08)	Reference	0.94 (0.89, 0.99)	0.83 (0.71, 0.97)	< 0.001	0.275
Multivariable adjusted^d^		1.11 (1.06, 1.17)	1.05 (1.02, 1.09)	Reference	0.94 (0.89, 0.99)	0.83 (0.71, 0.97)	< 0.001	0.228
RPS‐IsoP	1599							
Age adjusted		1.21 (1.03, 1.42)	1.08 (1.01, 1.16)	Reference	0.93 (0.88, 0.99)	0.90 (0.82, 0.99)	0.049	0.017
Multivariable adjusted^d^		1.22 (1.04, 1.43)	1.09 (1.02, 1.16)	Reference	0.93 (0.88, 0.99)	0.90 (0.82, 0.98)	0.044	0.014
In the SCCS								
5‐F_2t_‐IsoP	285							
Age adjusted		1.24 (1.05, 1.46)	1.13 (1.03, 1.23)	Reference	0.92 (0.85, 0.99)	0.97 (0.84, 1.13)	0.023	0.006
Multivariable adjusted^d^		1.38 (1.08, 1.76)	1.21 (1.04, 1.41)	Reference	0.84 (0.72, 0.97)	0.84 (0.67, 1.06)	0.024	0.007
15‐F_2t_‐IsoP	285							
Age adjusted		1.07 (0.96, 1.19)	1.02 (0.99, 1.05)	Reference	0.98 (0.94, 1.01)	0.96 (0.87, 1.06)	0.411	0.279
Multivariable adjusted^d^		1.10 (0.85, 1.42)	1.03 (0.95, 1.12)	Reference	0.95 (0.84, 1.08)	0.90 (0.72, 1.12)	0.579	0.527
15‐F_2t_‐IsoP‐M	285							
Age adjusted		1.07 (0.96, 1.20)	1.02 (0.98, 1.07)	Reference	0.99 (0.94, 1.05)	1.00 (0.83, 1.21)	0.449	0.260
Multivariable adjusted^d^		1.28 (0.96, 1.72)	1.11 (0.98, 1.27)	Reference	0.92 (0.83, 1.02)	0.93 (0.77, 1.13)	0.243	0.096
RPS‐IsoP	285							
Age adjusted		0.98 (0.70, 1.36)	0.99 (0.88, 1.13)	Reference	0.99 (0.91, 1.08)	0.96 (0.78, 1.17)	0.896	0.773
Multivariable adjusted^d^		1.00 (0.71, 1.40)	1.00 (0.88, 1.14)	Reference	0.98 (0.90, 1.07)	0.92 (0.74, 1.14)	0.733	0.734

Abbreviations: 15‐F_2t_‐IsoP, 15‐F_2t_‐Isoprostane; 15‐F_2t_‐IsoP‐M, 2,3‐Dinor‐5,6‐dihydro‐15‐F_2t_‐Isoprostane; 5‐F_2t_‐IsoP, 5‐F_2t_‐Isoprostane; CI, confidence interval; ng/mg Cr, nanograms per milligram of creatinine; OR, odds ratio; RPS‐IsoP, ratio of 15‐F_2t_‐IsoP‐M (metabolic product) to 15‐F_2t_‐IsoP (substrate); SCCS, Southern Community Cohort Study; SMHS, Shanghai Men's Health Study; SWHS, Shanghai Women's Health Study.

^a^ OR was estimated using a conditional logistic regression model with restricted cubic‐spline functions, using the 50th percentile of biomarkers in controls as the reference.

^b^ Cutoffs were based on the percentile distribution of biomarker levels in controls for **5‐F**_**2t**_ **‐IsoP** (ng/mg Cr): 1.15 (10th), 1.95 (30th), 2.79 (50th), 4.37 (70th), and 7.90 (90th) in the SWHS and SMHS; and 0.74 (10th), 1.18 (30th), 1.95 (50th), 3.08 (70th) and 6.01 (90th) in the SCCS. For **15‐F**_**2t**_ **‐IsoP** (ng/mg Cr): 0.64 (10th), 1.00 (30th), 1.20 (50th), 1.91 (70th), and 3.60 (90th) in the SWHS and SMHS; and 0.54 (10th), 0.83 (30th), 1.08 (50th), 1.50 (70th) and 2.89 (90th) in the SCCS. For **15‐F**_**2t**_ **‐IsoP‐M** (ng/mg Cr): 4.30 (10th), 7.27 (30th), 10.70 (50th), 15.88 (70th), and 25.14 (90th) in the SWHS and SMHS; and 6.81 (10th), 10.49 (30th), 14.75 (50th), 20.34 (70th) and 32.41 (90th) in the SCCS. For **RSP‐IsoP**: 272.6% (10th), 563.2% (30th), 810.3% (50th), 1140.9% (70th), and 1950.0% (90th) in the SWHS and SMHS; and 553.0% (10th), 1003.3% (30th), 1362.1% (50th), 1835.6% (70th) and 2586.6% (90th) in the SCCS.

^c^ Number of case–control pairs in the analysis after exclusion of batches with CV > 20%, with a case–control ratio of 1:1 in the SWHS, 1:1 in the SMHS, and 1:2 in the SCCS.

^d^ Multivariable adjusted for age at baseline, education, cigarette smoking, alcohol consumption, BMI, physical activity, regular use of vitamin supplements, regular use of aspirin and other nonsteroidal anti‐inflammatory drugs, Charlson comorbidity score, family history of colorectal cancer in first‐degree relatives, and total energy intake.

Comprehensive approaches to handling intra‐ and inter‐batch variations are detailed in Supporting Information Methods. Only biomarker measurements from assay batches with intra‐batch CV < 20% were included in the main analysis (83.4% for 15‐F_2t_‐IsoP, 91.7% for 5‐F_2t_‐IsoP, and 99.0% for 15‐F_2t_‐IsoP‐M in the Shanghai cohorts; 100% for all three markers in the SCCS). Inter‐batch effects were corrected by median‐centering biomarker values using the batch‐specific median of QC samples [20]. To assess potential selection bias from CV‐based exclusions, we compared baseline characteristics and follow‐up time distributions between included and excluded participants (Supporting Information Methods); the two groups were largely comparable (Tables S2 and S3). We also conducted sensitivity analyses by (1) retaining all samples regardless of CV and (2) repeating analyses without inter‐batch correction.

A composite OxS index was constructed using principal component analysis (PCA) of three markers: 8‐oxo‐dG (DNA), 8‐oxo‐Guo (RNA), and 5‐F_2t_‐IsoP (lipid). These markers were weakly to moderately correlated (*r* = 0.2–0.4 in Figure S3). The composite index was defined as the first principal component, which captures the largest shared variance across these measures, with the corresponding PCA loadings of 0.61 for 8‐oxo‐dG, 0.62 for 8‐oxo‐Guo, and 0.49 for 5‐F_2t_‐IsoP. All analyses were performed using R version 4.0.4 [21], with two‐sided *p* < 0.05 considered statistically significant.

## 3 Results

### 3.1 Participant Characteristics

The primary analysis included 1938 matched CRC case–control pairs from the SWHS and SMHS cohorts. The mean (SD) age at CRC diagnosis was 68.3 (9.3) years, with 53.4% being female. Cases and controls were generally comparable in demographic characteristics, lifestyle factors, and known CRC risk factors, except for a higher prevalence of CRC family history among cases (Table S4).

In the replication cohort SCCS, 285 CRC cases and 570 matched controls were included. The mean (SD) age at diagnosis was 55.1 (8.8) years, and 60.7% of cases were female. Baseline characteristics were largely similar between cases and controls (Table S5).

### 3.2 Time‐Dependent Inverse Association Between 5‐F_2t_‐IsoP and CRC Risk

In the Shanghai cohorts, urinary 5‐F_2t_‐IsoP levels were significantly lower in CRC cases compared to controls (*p* < 0.001; Table S6). A similar, though not statistically significant, trend was observed in the SCCS.

As shown in Table 1, higher baseline levels of 5‐F_2t_‐IsoP were associated with a significantly reduced risk of subsequent CRC in the Shanghai cohorts (*p* for overall association = 0.001). Multivariable‐adjusted ORs (95% CI) for CRC at the 10th and 90th percentiles of 5‐F_2t_‐IsoP, relative to the median, were 1.12 (1.04–1.21) and 0.84 (0.73–0.97), respectively. This inverse association was replicated in the SCCS (*p* = 0.02), with corresponding ORs (95% CI) of 1.38 (1.08–1.76) and 0.84 (0.67–1.06).

**FIGURE 1:**
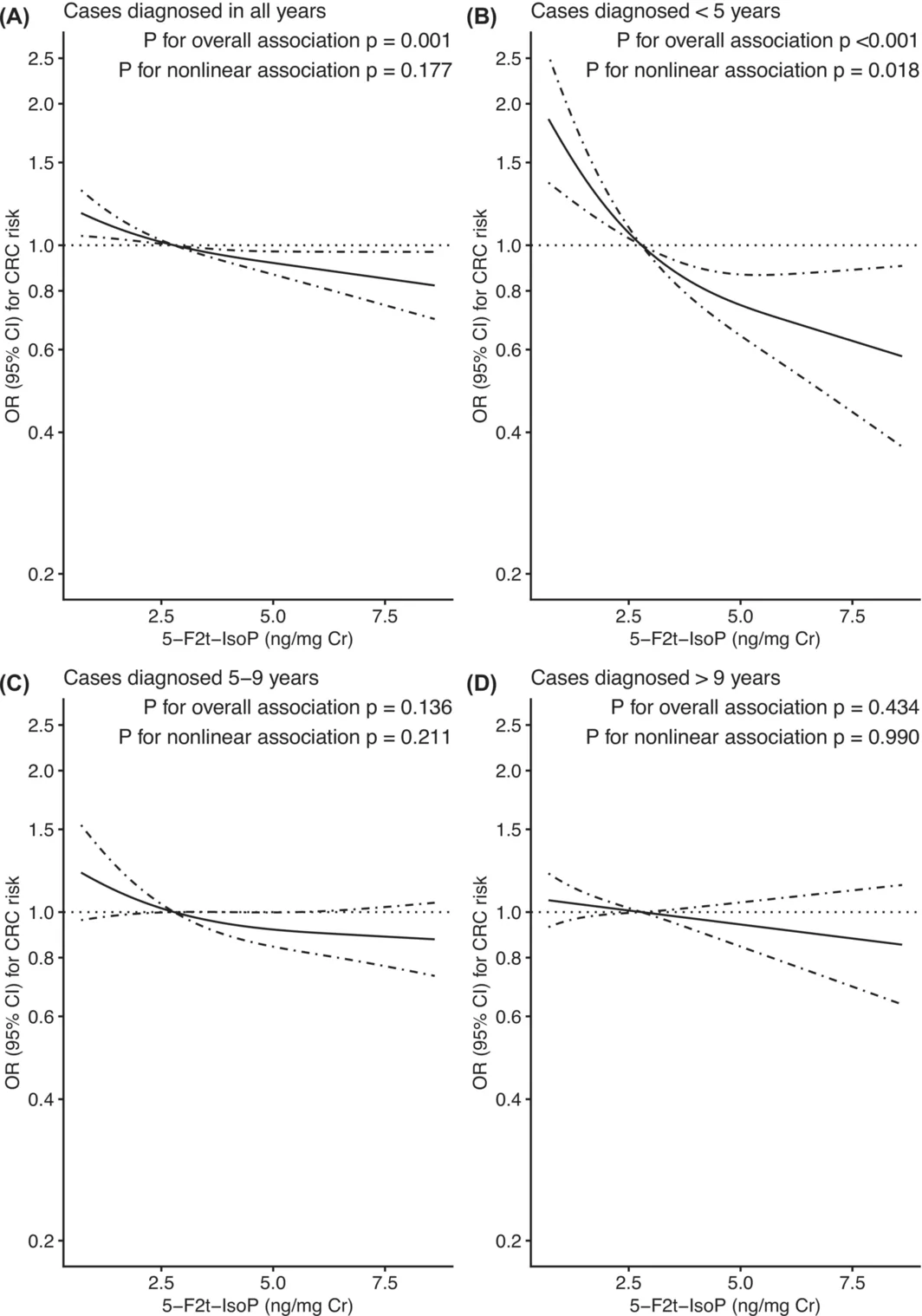
Smoothed plot for multivariable‐adjusted odds ratios for colorectal cancer risk according to urinary levels of 5‐F_2t_‐IsoP, by time interval from baseline to cancer diagnosis: Overall (A), < 5 years (B), 5–9 years (C), and > 9 years (D) in the SWHS and the SMHS. The OR was estimated using a conditional logistic regression model with restricted cubic spline functions, with the 50th percentile of biomarker levels in controls as the reference and adjusted for selected covariates as listed in the footnotes of Table 1. The OR values on the *y* axis are shown on a log scale. The solid line indicates the point estimate, and the dashed lines indicate the 95% CI. *p* for heterogeneity across the time interval groups was 0.037.

We further examined whether the inverse association varied over time. This analysis was motivated by our earlier findings that the relationship between DNA/RNA OxS markers and CRC risk was time‐dependent [1]. Given the limited number of cases in the SCCS (*n* = 285), time‐stratified analyses were restricted to the case–control pairs from the Shanghai cohorts (*n* = 1938). Stratified analyses revealed a significant time‐dependent pattern, with *p* for heterogeneity of 0.037 (Figure 1). Urinary 5‐F_2t_‐IsoP levels were strongly and inversely associated with CRC diagnosis within 5 years following enrollment (overall *p* < 0.0001). ORs (95% CI) at the 10th and 90th percentiles of 5‐F_2t_‐IsoP, relative to the median, were 1.57 (1.26–1.96) and 0.61 (0.42–0.89), respectively, indicating a 2.2‐fold difference in risk (Table 2). However, no significant associations were found for CRC diagnosed beyond 5 years.

**TABLE 2:** Associations between baseline urinary levels of F_2_‐IsoPs and subsequent risk of colorectal cancer by time interval between urine sample collection at baseline and cancer diagnosis during follow‐up in the SWHS and SMHS.

	Number of pairs^c^	OR (95% CI) for colorectal cancer risk relative to reference,^a^ by percentile distribution of biomarkers^b^					*p* for overall association	*p* for nonlinear association
		10th	30th	50th	70th	90th		
Cases diagnosed < 5 years after enrollment								
5‐F_2t_‐IsoP	387							
Age adjusted		1.51 (1.22, 1.86)	1.20 (1.10, 1.31)	Reference	0.80 (0.72, 0.89)	0.61 (0.42, 0.89)	< 0.001	0.036
Multivariable adjusted^d^		1.57 (1.26, 1.96)	1.22 (1.11, 1.33)	Reference	0.79 (0.71, 0.88)	0.61 (0.42, 0.89)	< 0.001	0.018
15‐F_2t_‐IsoP	367							
Age adjusted		1.06 (0.96, 1.18)	1.02 (0.99, 1.05)	Reference	0.96 (0.90, 1.03)	0.95 (0.82, 1.10)	0.522	0.311
Multivariable adjusted^d^		1.07 (0.96, 1.20)	1.02 (0.99, 1.05)	Reference	0.96 (0.90, 1.03)	0.96 (0.83, 1.12)	0.477	0.238
15‐F_2t_‐IsoP‐M	411							
Age adjusted		1.09 (0.97, 1.23)	1.03 (0.96, 1.10)	Reference	0.98 (0.87, 1.11)	0.96 (0.68, 1.35)	0.093	0.129
Multivariable adjusted^d^		1.08 (0.96, 1.23)	1.03 (0.95, 1.10)	Reference	0.99 (0.87, 1.12)	0.96 (0.67, 1.38)	0.137	0.145
RPS‐IsoP	349							
Age adjusted		1.04 (0.74, 1.46)	1.02 (0.89, 1.16)	Reference	0.99 (0.89, 1.10)	0.99 (0.84, 1.16)	0.747	0.798
Multivariable adjusted^d^		1.04 (0.73, 1.47)	1.01 (0.88, 1.16)	Reference	0.99 (0.89, 1.11)	0.99 (0.84, 1.17)	0.731	0.829
Cases diagnosed 5–9 years after enrollment								
5‐F_2t_‐IsoP	469							
Age adjusted		1.18 (1.01, 1.39)	1.08 (1.01, 1.15)	Reference	0.92 (0.86, 0.98)	0.87 (0.75, 1.01)	0.054	0.116
Multivariable adjusted^d^		1.15 (0.98, 1.36)	1.06 (0.99, 1.14)	Reference	0.93 (0.87, 1.00)	0.88 (0.75, 1.03)	0.136	0.211
15‐F_2t_‐IsoP	358							
Age adjusted		1.03 (0.92, 1.15)	1.01 (0.98, 1.04)	Reference	0.97 (0.91, 1.03)	0.91 (0.79, 1.03)	0.325	0.955
Multivariable adjusted^d^		1.01 (0.89, 1.14)	1.00 (0.97, 1.04)	Reference	0.98 (0.92, 1.05)	0.93 (0.82, 1.06)	0.529	0.849
15‐F_2t_‐IsoP‐M	539							
Age adjusted		1.10 (1.02, 1.20)	1.05 (1.00, 1.09)	Reference	0.94 (0.87, 1.02)	0.85 (0.68, 1.07)	0.058	0.450
Multivariable adjusted^d^		1.11 (1.02, 1.21)	1.05 (1.00, 1.10)	Reference	0.94 (0.87, 1.02)	0.85 (0.68, 1.07)	0.041	0.333
RPS‐IsoP	358							
Age adjusted		1.17 (0.85, 1.62)	1.07 (0.93, 1.22)	Reference	0.95 (0.85, 1.06)	0.92 (0.78, 1.09)	0.614	0.337
Multivariable adjusted^d^		1.27 (0.90, 1.78)	1.10 (0.96, 1.27)	Reference	0.92 (0.82, 1.04)	0.89 (0.74, 1.06)	0.382	0.171
Cases diagnosed > 9 years after enrollment								
5‐F_2t_‐IsoP	922							
Age adjusted		1.06 (0.98, 1.16)	1.03 (1.00, 1.07)	Reference	0.95 (0.88, 1.02)	0.84 (0.65, 1.08)	0.231	0.914
Multivariable adjusted^d^		1.05 (0.96, 1.14)	1.02 (0.99, 1.06)	Reference	0.96 (0.89, 1.03)	0.87 (0.67, 1.12)	0.434	0.990
15‐F_2t_‐IsoP	892							
Age adjusted		0.96 (0.89, 1.03)	0.99 (0.98, 1.01)	Reference	0.98 (0.94, 1.03)	0.90 (0.76, 1.06)	0.196	0.095
Multivariable adjusted^d^		0.96 (0.89, 1.03)	0.99 (0.97, 1.01)	Reference	0.98 (0.94, 1.03)	0.91 (0.77, 1.08)	0.242	0.107
15‐F_2t_‐IsoP‐M	969							
Age adjusted		1.14 (1.05, 1.24)	1.07 (1.02, 1.13)	Reference	0.90 (0.82, 0.99)	0.74 (0.57, 0.97)	0.008	0.998
Multivariable adjusted^d^		1.14 (1.04, 1.24)	1.07 (1.02, 1.13)	Reference	0.90 (0.82, 0.99)	0.75 (0.57, 0.98)	0.013	0.983
RPS‐IsoP	892							
Age adjusted		1.31 (1.06, 1.62)	1.13 (1.03, 1.24)	Reference	0.89 (0.81, 0.98)	0.84 (0.72, 0.98)	0.036	0.010
Multivariable adjusted^d^		1.31 (1.05, 1.62)	1.12 (1.02, 1.24)	Reference	0.89 (0.81, 0.98)	0.84 (0.72, 0.98)	0.046	0.013

Abbreviations: 5‐F_2t_‐IsoP, 5‐F_2t_‐Isoprostane; 15‐F_2t_‐IsoP, 15‐F_2t_‐Isoprostane; 15‐F_2t_‐IsoP‐M, 2,3‐Dinor‐5,6‐dihydro‐15‐F_2t_‐Isoprostane; RPS‐IsoP, ratio of 15‐F_2t_‐IsoP‐M (metabolic product) to 15‐F_2t_‐IsoP (substrate); CI, confidence interval; ng/mg Cr, nanograms per milligram of creatinine; OR, odds ratio; SMHS, Shanghai Men's Health Study; SWHS, Shanghai Women's Health Study.

^a^ OR was estimated using a conditional logistic regression model with restricted cubic‐spline functions, using the 50th percentile of biomarkers in controls as the reference.

^b^ Cutoffs were based on the percentile distribution of biomarker levels in controls as detailed in the footnotes of Table 1.

^c^ Number of case–control pairs in the analysis after excluding batches with CV > 20%.

^d^ Multivariable adjusted for age at baseline, education, cigarette smoking, alcohol consumption, BMI, physical activity, regular use of vitamin supplements, regular use of aspirin and other nonsteroidal anti‐inflammatory drugs, Charlson comorbidity score, family history of colorectal cancer in first‐degree relatives, and total energy intake.

### 3.3 Null Association Between 15‐F_2t_‐IsoP and CRC Risk

Urinary 15‐F_2t_‐IsoP levels did not differ significantly between CRC cases and controls in either the primary or replication cohorts (Table S6). Accordingly, no significant associations with overall CRC risk or in time‐stratified analyses were observed (Tables 1 and 2; Figure S4).

### 3.4 Association Between 15‐F_2t_‐IsoP‐M and CRC Risk

In the Shanghai cohorts, urinary levels of 15‐F_2t_‐IsoP‐M, unlike unmetabolized 15‐F_2t_‐IsoP, were significantly lower in CRC cases than in controls (*p* = 0.01; Table S6). Multivariable‐adjusted ORs (95% CI) for CRC risk at the 10th and 90th percentiles of 15‐F_2t_‐IsoP‐M, relative to the median, were 1.11 (1.06–1.17) and 0.83 (0.71–0.97), respectively, with overall *p* < 0.0001 (Table 1).

The ratio of metabolic product 15‐F_2t_‐IsoP‐M to its substrate 15‐F_2t_‐IsoP (RPS‐IsoP) was also lower in CRC cases compared to controls (*p* = 0.04). A higher RPS‐IsoP was associated with significantly reduced CRC risk, with the corresponding ORs (95% CI) for CRC risk being 1.22 (1.04–1.43) and 0.90 (0.82–0.98) at the 10th and 90th percentiles of RPS‐IsoP, respectively (Table 1).

Interestingly, while the inverse association between 5‐F_2t_‐IsoP and CRC risk diminished over time, associations for 15‐F_2t_‐IsoP‐M and RPS‐IsoP became stronger with longer follow‐up (Table 2 and Figures S5 and S6). For example, 15‐F_2t_‐IsoP‐M was not significantly associated with CRC risk for cancers diagnosed within 5 years (*p* = 0.14), but the association became significant for diagnoses at 5–9 years (*p* = 0.04) and > 9 years (*p* = 0.01). However, no significant associations were observed with overall CRC risk in the SCCS.

### 3.5 Sensitivity Analyses

The time‐dependent inverse association between 5‐F_2t_‐IsoP and CRC risk was consistent across subgroups defined by sex, race, and tumor location, although statistically not significant in some subgroups due to limited sample sizes (Tables S7–S11). To evaluate the impact of inter‐batch assay variations, we compared results obtained with and without inter‐batch correction. Findings were robust across sensitivity analyses restricted to assay batches with CV < 20% with and without correction (Tables 1 and 2 and Tables S12 and S13) and in analyses including all samples regardless of CV (Tables S14 and S15).

### 3.6 Composite OxS Index and CRC Risk

**FIGURE 2:**
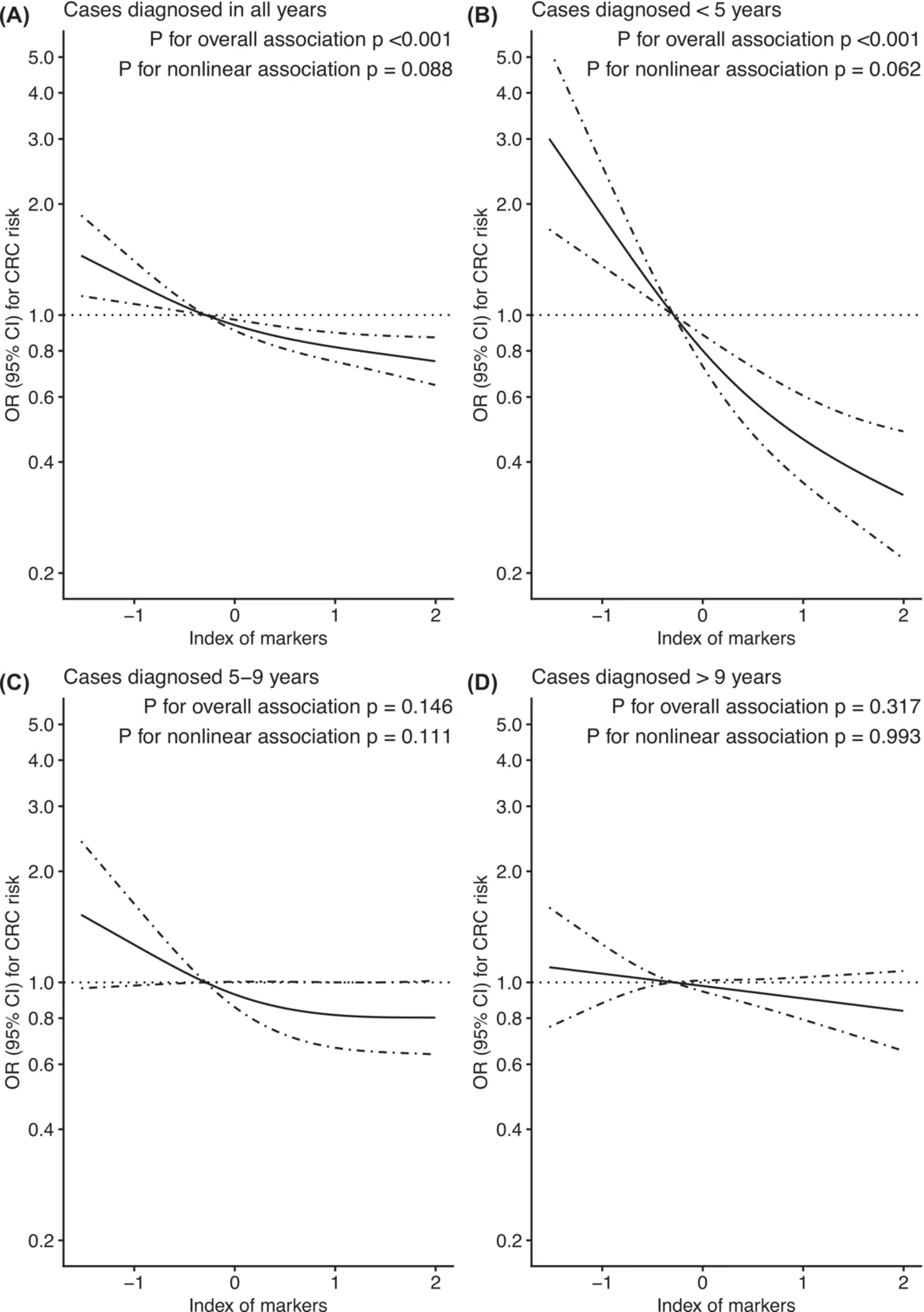
Smoothed plot for multivariable‐adjusted odds ratios for colorectal cancer risk according to the composite index of oxidative stress markers, by time interval from baseline to cancer diagnosis: Overall (A), < 5 years (B), 5–9 years (C), and > 9 years (D) in the SWHS and SMHS. The OR was estimated using a conditional logistic regression model with restricted cubic spline functions, with the 50th percentile of the OxS index in controls as the reference and adjusted for selected covariates as listed in the footnotes of Table 1. The index was constructed using principal component analysis for 8‐oxo‐dG (DNA), 8‐oxo‐Guo (RNA), and 5‐F_2t_‐IsoP (lipid) OxS markers. The OR values on the *y* axis are shown on a log scale. The solid line indicates the point estimate, and the dashed lines indicate the 95% CI. *p* for heterogeneity across the time interval groups was < 0.001.

A composite OxS index incorporating 8‐oxo‐dG (DNA), 8‐oxo‐Guo (RNA), and 5‐F_2t_‐IsoP (lipid) markers was inversely associated with CRC risk in both the Shanghai and US cohorts (Table S16). This association varied by time from enrollment to diagnosis (Figure 2). In addition, the composite index outperformed individual biomarkers in predicting CRC risk (Table 3). For CRC diagnosed within 5 years, ORs (95% CI) at the 10th and 90th percentiles of the index, relative to the median, were 2.11 (1.44–3.09) and 0.36 (0.25–0.51), respectively. This reflects an approximately 4‐fold difference in risk between the two groups, compared to 2‐ to 3‐fold differences in risk when using individual markers.

**TABLE 3:** Multivariable‐adjusted odds ratio and confidence intervals for colorectal cancer diagnosed within 5 years of enrollment: Comparison of oxidative stress index with individual markers in the SWHS and SMHS.

	OR (95% CI) for colorectal cancer risk relative to reference,^a^ by percentile distribution of biomarkers^b^					*p* for overall association	*p* for nonlinear association	Fold difference in OR b/w the 10th and the 90th
	10th	30th	50th	70th	90th			
Index of OxS markers^c^	2.11 (1.44, 3.09)	1.43 (1.20, 1.70)	Reference	0.62 (0.52, 0.75)	0.36 (0.25, 0.51)	< 0.001	0.062	3.89
5‐F_2t_‐IsoP	1.57 (1.26, 1.96)	1.22 (1.11, 1.33)	Reference	0.79 (0.71, 0.88)	0.61 (0.42, 0.89)	< 0.001	0.018	2.20
8‐oxo‐dG^d^	1.87 (1.39, 2.53)	1.34 (1.17, 1.54)	Reference	0.70 (0.61, 0.80)	0.48 (0.37, 0.63)	< 0.001	0.02	2.95
8‐oxo‐Guo^d^	1.46 (1.07, 1.98)	1.19 (1.04, 1.37)	Reference	0.80 (0.69, 0.93)	0.57 (0.42, 0.76)	< 0.001	0.26	2.21

Abbreviations: 5‐F_2t_‐IsoP, 5‐F_2t_‐Isoprostane; 8‐oxo‐dG, 8‐oxo‐7,8‐dihydro‐2′‐deoxyguanosine; 8‐oxo‐Guo, 7,8‐dihydro‐8‐oxo‐guanosine; CI, confidence interval; OR, odds ratio; SMHS, Shanghai Men's Health Study; SWHS, Shanghai Women's Health Study.

^a^ OR was estimated using a conditional logistic regression model with restricted cubic spline functions, with the 50th percentile of biomarker in controls as the reference, and adjusted for age at baseline, education, cigarette smoking, alcohol consumption, BMI, physical activity, regular use of vitamin supplements, regular use of aspirin and other nonsteroidal anti‐inflammatory drugs, Charlson comorbidity score, family history of colorectal cancer in first‐degree relatives, and total energy intake.

^b^ Cutoffs were based on the percentile distribution of biomarker levels in controls as detailed in the footnotes of Table 1.

^c^ The index was constructed using principal component analysis for 8‐oxo‐dG (DNA), 8‐oxo‐Guo (RNA) and 5‐F_2t_‐IsoP (lipid) OxS markers.

^d^ Details have been reported in MedRxiv. 2025; doi: 10.1101/2025.01.21.25320898.

## 4 Discussion

This study presents new evidence supporting a time‐dependent inverse association between systemic OxS and CRC risk. The observed inverse relationship between urinary lipid peroxidation markers, especially 5‐F_2t_‐IsoP, and CRC risk complements our previous findings on DNA and RNA OxS markers [1], suggesting a consistent pattern of declining systemic OxS as CRC progresses. Reduced systemic OxS was associated with significantly increased CRC risk during the later stages of development. Importantly, this pattern was consistently observed across three independent cohorts, despite differences in baseline characteristics such as racial/ethnic composition and risk profiles. In addition, the composite OxS index, incorporating DNA, RNA, and lipid oxidation markers, demonstrated superior predictive performance compared to individual markers. This suggests that multi‐target OxS profiling may better capture the complex interplay of oxidative damage across macromolecular domains in CRC development.

Prospective investigations into the relationship between systemic lipid peroxidation and CRC risk have been very limited. Two nested case–control studies reported positive associations between higher levels of serum hydroperoxides or oxidized low‐density lipoprotein and increased CRC risk [22, 23]. However, these two markers are not considered reliable indicators of systemic OxS [24, 25]. In contrast, a null association between urinary F_2_‐IsoPs and colorectal adenoma risk was reported in a cohort study after 10 years of follow‐up [26]. Most prior studies have employed cross‐sectional designs [27, 28, 29], comparing cancer‐free individuals to cancer patients with biological samples collected after diagnosis and often during cancer treatment. This approach is suboptimal for investigating early molecular events; and cancer treatment can elevate systemic OxS, potentially biasing results. Additionally, many of these studies were limited by small sample sizes (often < 100 cases) [27, 28, 29] and relied on ELISA‐based methods for OxS quantification [30]. More importantly, few studies have considered this potential time‐dependent nature of the OxS and cancer relationship. To our knowledge, this is the first population‐based study to examine the dynamic relationship between systemic lipid peroxidation and CRC risk over time.

Given that more than two decades may elapse between the initiation and clinical manifestation of cancer in humans [31], individuals diagnosed within the first 5 years following enrollment were very likely already in the later stage of cancer development at the time of enrollment. Accordingly, the findings of this study, together with our recent report on DNA and RNA OxS markers [1], suggest that the observed reduction in systemic OxS likely occurs during the late stage of colorectal tumorigenesis, potentially coinciding with metabolic reprogramming at the primary cancer site. Therefore, this study extends observations from experimental models at the cellular levels to the systemic level in a population‐based setting. Because the signaling pathways underlying these systemic alterations remain poorly understood, and given that this is a new observation, further investigation into the temporal dynamics of OxS in CRC development is warranted.

The time‐dependent inverse association with CRC risk was observed for 5‐F_2t_‐IsoP, but not for 15‐F_2t_‐IsoP. Although both 5‐F_2t_‐IsoP and 15‐F_2t_‐IsoP in urine are used to assess systemic lipid peroxidation, their biochemical origins differ in ways that may affect their utility as prospective risk markers of cancer. Unlike 5‐F_2t_‐IsoP, which is formed exclusively through free‐radical–mediated oxidation of arachidonic acid [15], 15‐F_2t_‐IsoP can also be generated enzymatically via the COX pathway [16, 17, 18]. This dual origin may reduce its specificity as a systemic OxS biomarker in disease settings characterized by chronic inflammation and COX‐2 upregulation, such as colorectal carcinogenesis. Increased enzymatic COX activity during tumor development may introduce inflammation‐related “noise” into 15‐F_2t_‐IsoP measurements, thereby masking the inverse association between OxS and late‐stage CRC progression. Therefore, lipid peroxidation markers that integrate both enzymatic and non‐enzymatic pathways may be less informative for prospective risk assessment. In contrast, biomarkers with greater biochemical specificity for free‐radical oxidation, such as 5‐F_2t_‐IsoP, may provide clearer insights into systemic OxS dynamics during advanced stages of CRC development.

Interestingly, 15‐F_2t_‐IsoP‐M, a β‐oxidation metabolite of 15‐F_2t_‐IsoP, and the corresponding product‐to‐substrate ratio (RPS‐IsoP) were also inversely associated with CRC risk, most notably among cases diagnosed ≥ 5 years after urine collection. In contrast, unmetabolized 15‐F_2t_‐IsoP showed no association. The β‐oxidation of 15‐F_2t_‐IsoP is known to occur predominantly in peroxisomes [32, 33]. RPS‐IsoP likely reflects both upstream lipid peroxidation and downstream metabolic processing of oxidized lipids. In this context, higher RPS‐IsoP may not simply indicate lower systemic OxS; rather, it may represent a metabolic phenotype with more efficient handling of lipid peroxidation products, potentially reducing their downstream effects on redox‐sensitive signaling pathways. During earlier stages of colorectal tumorigenesis, when tumor‐induced metabolic reprogramming is likely limited, such enhanced oxidative lipid metabolism could contribute to a more favorable systemic environment. To our knowledge, RPS‐IsoP and its time‐dependent relationship with CRC risk have not been evaluated previously in population‐based studies. Therefore, these findings should be considered hypothesis‐generating and warrant replication and mechanistic investigation.

Our time‐dependent findings, particularly the stronger inverse associations observed within 5 years of diagnosis, raise the possibility of reverse causation. Rather than indicating a protective effect of lower systemic OxS, reduced OxS during this period may reflect tumor‐ or host‐driven adaptations to the metabolic and OxS of an undiagnosed, advancing malignancy. Cancer cells are typically more vulnerable to high OxS than normal cells. Increased OxS, if not controlled, can impair essential macromolecules, triggering senescence or apoptosis [34, 35]. Experimental models suggest that cancer cells subjected to high metabolic stress may undergo metabolic reprogramming [36, 37, 38], activating a variety of antioxidant defense mechanisms to mitigate OxS [4, 37, 38, 39], to promote tumor growth and metastasis [3, 40, 41]. From this perspective, the < 5‐year association may indicate that systemic OxS measures capture host‐tumor interactions during late‐phase development or progression, rather than initiation risk. This interpretation, although biologically plausible, requires further investigation.

Our study has several notable strengths, including the two‐phase approach involving both the primary and replication analyses, the use of specific biomarkers quantified by mass spectrometric‐based methods, and comprehensive analytical approaches to evaluate the association in a time‐dependent manner. Unlike previous studies that were often constrained by small sample sizes or retrospective designs, the large sample size and long‐term follow‐up in the SWHS and SMHS allowed us to investigate alterations in systemic lipid peroxidation with CRC progression, although the number of cases in the SCCS was insufficient for evaluating this time‐varying association.

Some limitations should be acknowledged. First, the urine samples were collected at a single time point. However, we previously demonstrated that levels of both F_2_‐IsoPs and 15‐F_2t_‐IsoP‐M remained stable over time [42], suggesting that a single measurement provides a reasonably reliable estimate of long‐term OxS status among healthy individuals. Second, residual confounding by unmeasured or imperfectly measured factors, such as unrecorded lifestyle or dietary changes preceding clinical diagnosis, cannot be entirely excluded, despite extensive covariate adjustment. However, our prior investigations suggest that lifestyle, dietary, and behavioral factors explain only a small proportion of the variability in systemic OxS [43, 44, 45, 46, 47]. Third, urine samples were available for only 27.6% of SCCS participants. However, baseline characteristics were comparable between those with and without urine samples, with SMD ranging from 0.02 to 0.11 [1]. Finally, although the overall inverse association between urinary 5‐F_2t_‐IsoP and CRC risk was directionally consistent across cohorts, the time‐stratified pattern was identified in the two Shanghai cohorts, where sample size supported lag‐specific analyses. In SCCS, limited case numbers precluded comparable time‐stratified modeling; therefore, SCCS provides replication of the overall association but does not permit evaluation of the stage‐dependent association. Additional studies in diverse populations will be needed to assess whether the time‐dependent association generalizes across different ethnic populations. Moreover, the generalizability of this finding to other cancer types remains uncertain, underscoring the need for further research.

## 5 Conclusion

This study, together with our recent report [1], suggests that reduced systemic OxS may contribute to increased CRC risk in its late stage of development. This aligns with previous RCTs indicating that lowering systemic OxS through antioxidant supplementation may pose risks to high‐risk individuals. Given the widespread use of such supplements globally, these findings underscore the need for caution in recommending antioxidant interventions, particularly for individuals at elevated cancer risk. Further studies are warranted to validate these observations and to elucidate the temporal dynamics and mechanistic underpinnings of systemic OxS in cancer development.

## Author Contributions

**Gong Yang:** conceptualization, data curation, supervision, resources, project administration, writing – review and editing, writing – original draft, funding acquisition, investigation. **Ginger L. Milne:** conceptualization, investigation, writing – review and editing, project administration, data curation. **Marina S. Nogueira:** data curation, writing – review and editing, investigation. **Yu‐Tang Gao:** data curation, investigation, resources, writing – review and editing. **Qing Lan:** resources, writing – review and editing, investigation, data curation. **Haoyang Yi:** formal analysis, investigation, writing – review and editing. **Xiao‐Ou Shu:** data curation, resources, investigation, writing – review and editing, funding acquisition. **Wei Zheng:** investigation, funding acquisition, resources, writing – review and editing, data curation. **Qingxia Chen:** conceptualization, data curation, formal analysis, investigation, funding acquisition, writing – review and editing.

## Funding

This work was supported by the Division of Cancer Prevention, National Institutes of Health (grant numbers R01CA237895, R01CA122364, UM1CA182910, UM1CA173640, and U01CA202979) and partially supported by the Vanderbilt‐Ingram Cancer Center (P30CA068485). The funders had no role in the design and conduct of the study; collection, management, analysis, or interpretation of the data; preparation, review, or approval of the manuscript; or decision to submit the manuscript for publication.

## Ethics Statement

All participants provided written informed consent. This study was approved by Vanderbilt University Medical Center Institutional Review Board for human research (IRB #191648).

## Conflicts of Interest

The authors declare no conflicts of interest.

## Supporting information


Supporting Information: Methods:
**Table S1:** Deviance residual diagnostic summary for urinary 5‐F_2t_‐IsoP by follow‐up time.
**Table S2:** Distributions of baseline characteristics and follow‐up time by 5‐F_2t_‐IsoP CV categories in SMHS controls.
**Table S3:** Distributions of baseline characteristics and follow‐up time by 5‐F_2t_‐IsoP CV categories in SMHS cases.
**Table S4:** Baseline characteristics of colorectal cancer cases and matched controls in the SWHS and the SMHS.
**Table S5:** Baseline characteristics of colorectal cancer cases and matched controls in the SCCS.
**Table S6:** Urinary F_2_‐IsoP levels in colorectal cancer cases and controls.
**Table S7:** Associations between baseline urinary F_2_‐IsoP levels and subsequent colorectal cancer risk by sex in the SWHS and SMHS.
**Table S8:** Associations between baseline urinary F_2_‐IsoP levels and subsequent colorectal cancer risk by race in the SCCS.
**Table S9:** Associations between baseline urinary F_2_‐IsoP levels and subsequent colorectal cancer risk by time interval between urine sample collection at baseline and cancer diagnosis during follow‐up in the SWHS.
**Table S10:** Associations between baseline urinary F_2_‐IsoP levels and subsequent colorectal cancer risk by time interval between urine sample collection at baseline and cancer diagnosis during follow‐up in the SMHS.
**Table S11:** Associations between baseline urinary F_2_‐IsoP levels and subsequent colorectal cancer risk in the SWHS and the SMHS, stratified by anatomical sites and time interval between urine sample collection at baseline and cancer diagnosis during follow‐up.
**Table S12:** Associations between baseline urinary F_2_‐IsoP levels and subsequent colorectal cancer risk: analyses restricted to batches with CV < 20% and without inter‐batch correction.
**Table S13:** Associations between baseline urinary F_2_‐IsoP levels and subsequent colorectal cancer risk, stratified by follow‐up time: analyses restricted to batches with CV < 20% and without inter‐batch correction.
**Table S14:** Associations between baseline urinary F_2_‐IsoP levels and subsequent colorectal cancer risk: analyses including all participants without inter‐batch correction.
**Table S15:** Associations between baseline urinary F_2_‐IsoP levels and subsequent colorectal cancer risk, stratified by follow‐up time: analyses including all participants from the SWHS and SMHS, without inter‐batch correction.
**Table S16:** Associations between a composite index of oxidative stress markers and subsequent risk of colorectal cancer.
**Figure S1:** Participant flow chart.
**Figure S2:** Distribution of follow‑up time from enrollment to CRC diagnosis.
**Figure S3:** Correlations among oxidative stress markers of nucleic acids and lipids in urine.
**Figure S4:** Smoothed plot of multivariable‐adjusted ORs for colorectal cancer risk by urinary 15‐F_2t_‐IsoP levels and time interval from baseline to cancer diagnosis.
**Figure S5:** Smoothed plot of multivariable‐adjusted ORs for colorectal cancer risk by urinary 15‐F_2t_‐IsoP‐M levels and by time interval from baseline to cancer diagnosis.
**Figure S6:** Smoothed plot of multivariable‐adjusted ORs for colorectal cancer risk by RPS‐IsoP and time interval from baseline to cancer diagnosis.

## Data Availability

Information and de‐identified data that support the findings of this study are available from the corresponding author upon reasonable request, pending approval by the data management and sharing committees of the SWHS, SMHS, and SCCS. Code book is publicly available on https://swhs‐smhs.app.vumc.org/ for the SWHS and SMHS and https://www.southerncommunitystudy.org/ for the SCCS. R code used in this analysis can be found at GitHub https://github.com/hyi414/ISOP_CRC_Risk/tree/main.
