# Supplementary material for: Systemic Lipid Peroxidation and Colorectal Cancer Risk: A Time‐Varying Relationship
Source: Int J Cancer. 2026 Jun 15;159(9):2170–83. doi: 10.1002/ijc.70585 (PMC13547988; doi:10.1002/ijc.70585)
Supplement: Supplementary file 1 [file IJC-159-2170-s001.docx]

**Manuscript title:** Systemic Lipid Peroxidation and Colorectal Cancer Risk: A Time-Varying Relationship

**Author list:** Gong Yang, Ginger L. Milne, Marina S. Nogueira, Yu-Tang Gao, Qing Lan, Haoyang Yi, Xiao-Ou Shu, Wei Zheng, Qingxia Chen

**Supplemental Online Content**

1. **Supplemental Methods**
   1.1 Cohort follow-up
   1.2 Laboratory measurements
   1.3 Statistical analysis: Model adequacy assessment
   1.4 Statistical analysis: Correction of batch effects and sensitivity analysis
   1.5 Statistical analysis: CV-based exclusion, potential selection bias, and sensitivity analysis
2. **Supplemental Table 1**. Deviance residual diagnostic summary for urinary 5-F_2t_-IsoP by follow-up time
3. **Supplemental Table 2.** Distributions of baseline characteristics and follow-up time by 5-F_2t_-IsoP CV categories in SMHS controls
4. **Supplemental Table 3.** Distributions of baseline characteristics and follow-up time by 5-F_2t_-IsoP CV categories in SMHS cases
5. **Supplemental Table 4.** Baseline characteristics of colorectal cancer cases and matched controls in the SWHS and the SMHS
6. **Supplemental Table 5.** Baseline characteristics of colorectal cancer cases and matched controls in the SCCS
7. **Supplemental Table 6.** Urinary F_2_-IsoP levels in colorectal cancer cases and controls
8. **Supplemental Table 7.** Associations between baseline urinary F_2_-IsoP levels and subsequent colorectal cancer risk by sex in the SWHS and SMHS
9. **Supplemental Table 8.** Associations between baseline urinary F_2_-IsoP levels and subsequent colorectal cancer risk by race in the SCCS
10. **Supplemental Table 9.** Associations between baseline urinary F_2_-IsoP levels and subsequent colorectal cancer risk by time interval between urine sample collection at baseline and cancer diagnosis during follow-up in the SWHS
11. **Supplemental Table 10.** Associations between baseline urinary F_2_-IsoP levels and subsequent colorectal cancer risk by time interval between urine sample collection at baseline and cancer diagnosis during follow-up in the SMHS
12. **Supplemental Table 11.** Associations between baseline urinary F_2_-IsoP levels and subsequent colorectal cancer risk in the SWHS and the SMHS, stratified by anatomical sites and time interval between urine sample collection at baseline and cancer diagnosis during follow-up
13. **Supplemental Table 12.** Associations between baseline urinary F_2_-IsoP levels and subsequent colorectal cancer risk: analyses restricted to batches with CV <20% and without inter-batch correction
14. **Supplemental Table 13.** Associations between baseline urinary F_2_-IsoP levels and subsequent colorectal cancer risk, stratified by follow-up time: analyses restricted to batches with CV <20% and without inter-batch correction
15. **Supplemental Table 14.** Associations between baseline urinary F_2_-IsoP levels and subsequent colorectal cancer risk: analyses including all participants without inter-batch correction
16. **Supplemental Table 15.** Associations between baseline urinary F_2_-IsoP levels and subsequent colorectal cancer risk, stratified by follow-up time: analyses including all participants from the SWHS and SMHS, without inter-batch correction
17. **Supplemental Table 16.** Associations between a composite index of oxidative stress markers and subsequent risk of colorectal cancer
18. **Supplemental Figure 1.** Participant Flow Chart
19. **Supplemental Figure 2.** Distribution of follow‑up time from enrollment to CRC diagnosis
20. **Supplemental Figure 3.** Correlations among oxidative stress markers of nucleic acids and lipids in urine
21. **Supplemental Figure 4.** Smoothed plot of multivariable-adjusted ORs for colorectal cancer risk by urinary 15-F_2t_-IsoP levels and time interval from baseline to cancer diagnosis
22. **Supplemental Figure 5.** Smoothed plot of multivariable-adjusted ORs for colorectal cancer risk by urinary 15-F_2t_-IsoP-M levels and by time interval from baseline to cancer diagnosis
23. **Supplemental Figure 6.** Smoothed plot of multivariable-adjusted ORs for colorectal cancer risk by RPS-IsoP and time interval from baseline to cancer diagnosis
1. **Supplemental Methods**
   1.1. **Cohort follow-up**

Cohort members in SWHS and SMHS have been followed for the occurrence of cancer through a combination of repeated home visits (every 2-4 years) and annual record linkage to Shanghai Tumor Registry and Shanghai Vital Statistics. The loss to follow-up was very low in SMHS (0.04%) and SWHS (0.62%). These individuals had no follow-up time (i.e., follow-up duration = 0) and were excluded from the cohort at baseline. A total of 409 participants fell into this category. The administrative end of this study was on June 30, 2017, for both Shanghai cohorts. CRC diagnosis was verified via reviewing medical records.

Incident cancer cases in SCCS were determined through linkage with 12 US state cancer registries and National Death Index mortality records. Therefore, the follow-up for CRC event was virtually complete. The administrative end of this study in SCCS was December 31, 2018.

The distribution of follow‑up time from enrollment to CRC diagnosis is shown in Supplemental Figure 2. A small number of cases were diagnosed within the first month after urine collection (SWHS: 1; SMHS: 2; SCCS: 2).

1.2. **Laboratory measurements**

A stock solution of the isotopically-labeled internal standard ([^2^H_4_]-15-F_2t_-IsoP, Item No. 316350, Cayman Chemical, Ann Arbor, MI USA) was prepared in ethanol. The concentration of the stock solution was determined by comparison with the 15-F_2t_-IsoP MaxSpec© standard (Item No. 25903, Cayman Chemical, Ann Arbor, MI USA).

Urine was thawed on ice. A 0.200mL aliquot was diluted with 0.400mL of a solution of 0.1% formic acid/methanol (95/5, v/v) and 0.040mL 0.1N HCl. The solution was vortexed to mix and to [^2^H_4_]-15-F_2t_-IsoP (1.8ng) was added to each sample. The samples were again mixed and the pH of each was adjusted to pH3 with 0.1N HCl, if required.

The samples were purified by extraction on a Waters HLB 96-well micro-elution plate (Waters Corporation, Milford, MA USA). Sample wells were first washed with methanol (0.200mL x 2) followed by 25% methanol in water (0.200mL x 2). The sample was then loaded on the matrix and washed with 0.400mL of a solution of 0.1% formic acid/methanol (95/5, v/v). The wells were then washed with 0.200mL hexanes. Isoprostanes and metabolites were eluted from the plate with 0.030mL 2-propanol/acetonitrile (50/50, v/v) into a 96-well collection plate containing 0.030mL water in each well.

Samples were analyzed on a Waters Xevo TQ-XS triple quadrupole mass spectrometer connected to a Waters Acquity I-Class UPLC (Waters Corp., Milford, MA USA). Separation of analytes was obtained using a Waters BEH C18 UPLC column (1.0 x 100mm, 1.8mm) with mobile phase A being 0.01% formic acid in water and mobile phase B acetonitrile. Isoprostanes and metabolites were separated using a gradient elution beginning with 20% B going to 95% B at a flow rate of 0.100mL/min (see Gradient Table). The mass spectrometer was operated using multiple reaction monitoring (MRM) in the negative ion mode with argon as the collision gas. Instrument control and data acquisition utilized MassLynx V4.2; integration and quantitation used TargetLynx 4.2. The limit of detection is 0.050 ng/mL; CV, <8%; accuracy, 94%.

| **UPLC Conditions** | |  |  |  |
| --- | --- | --- | --- | --- |
| **Column Temp** | 35^o^C |  |  |  |
| **Sample Temp** | 8^o^C |  |  |  |
| **Inj Mode** | Partial Loop |  |  |  |
| **Inj Loop** | 10μL |  |  |  |
| **Inj Vol** | 5μL |  |  |  |
|  |  |  |  |  |
| **UPLC Gradient** |  |  |  |  |
| **Time (min)** | **Flow Rate (mL/min)** | **%A** | **%B** | **Curve** |
| Initial | 0.100 | 80 | 20 | Initial |
| 0.10 | 0.100 | 80 | 20 | 6 |
| 5.00 | 0.100 | 60 | 40 | 6 |
| 6.00 | 0.100 | 5 | 95 | 6 |
| 8.50 | 0.100 | 5 | 95 | 6 |
| 8.60 | 0.100 | 70 | 30 | 6 |
| 11.00 | 0.100 | 70 | 30 | 6 |

| **Mass Spectrometer Settings** | |
| --- | --- |
| **Operation Mode** | ESI negative |
| **Capillary** | 1 kV |
| **Desolvation Temp** | 500 V |
| **Desolvation Gas** | 1000 L/hr |
| **Cone Gas** | 150 L/hr |
| **Nebuliser** | 7 bar |

| **Mass-to-charge ratios for F_2_-IsoPs** | | | | | | |  |  |
| --- | --- | --- | --- | --- | --- | --- | --- | --- |
| **Analyte** | **Internal Std** | **Precursor**  **Ion**  **(m/z)** | **Product**  **Ion (m/z)** | **Cone**  **(Volts)** | **Collision**  **Energy** | **Dwell time**  **(seconds)** |  |  |
| 2,3-dinor-5,6-dihydro-15-F_2t_-IsoP | 15-F_2t_-IsoP-d_4_ | 327.1 | 283.1 | 40 | 24 | 0.022 |  |  |
| 15-series-F_2_-IsoPs | 15-F_2t_-IsoP-d_4_ | 353.1 | 193.1 | 40 | 24 | 0.022 |  |  |
| 5-series-F_2_-IsoPs | 15-F_2t_-IsoP-d_4_ | 353.102 | 115.1 | 40 | 24 | 0.022 |  |  |

1.3. **Statistical analysis: Model adequacy assessment**

We assessed model adequacy for the conditional logistic regression models using deviance residual diagnostics. Using 5‑F_2t_‑IsoP as an illustrative example, residuals were centered near zero without systematic patterns across biomarker values, and slopes from regressing residuals on fitted 5‑F_2t_‑IsoP values were not significantly different from zero (all P ≥0.55), indicating no material lack of fit for the spline specification of 5‑F_2t_‑IsoP in the conditional logistic models. Diagnostic summaries are provided in **Supplemental Table 1**.

1.4. **Statistical analysis: Correction of batch effects and sensitivity analysis**

Both intra-batch and inter-batch variabilities can contribute to uncertainty in research results. In this study, test samples were processed in five sequential sampling groups, each comprising several hundred to over a thousand samples. Within each intra-day batch, approximately 30 samples including four quality control (QC) samples, were analyzed. The entire measurement spanned approximately 2 years, during which a minor refinement in the laboratory method was implemented.

To account for potential sources of variability, we incorporated intra-batch and inter-batch variation, QC assessments, and methodological refinements into our downstream analyses.

Intra-batch variability was assessed by calculating the coefficient of variation (CV) for QC samples within each batch (n = 4, representing ~13% of samples per batch). In our laboratory, CVs for selected analytes typically remain below 8%. Batches with CVs exceeding 20% were excluded from the primary analysis.

To minimize inter-batch variation, case-control pairs were analyzed within the same assay batch with a random and blind arrangement. Conditional logistic regression models stratified by matched sets were employed, which helped mitigate the influence of inter-batch effects.

We applied an inter-batch correction procedure to account for batch-to-batch variation. A batch-specific QC-based ratio (QBR) was calculated as the ratio of the median QC value within each batch to the overall median across all batches. Biomarker values were then corrected by dividing by the corresponding QBR. Since two QC samples were used across batches, an additional correction factor (QR) was introduced to account for differences between them. QR was defined as the ratio of the median of the first QC sample to that of the second QC sample, with QR₁ set to 1.

This two-step transformation leads to the following correction algorithm. Let

$Normalized_{ik}=Sample_{ik} \times QBR_{ik}\times QR_{k}$,

$$QBR_{ik}=\frac{X_{.k}}{X_{ik}}, i = 1\ldots n_{k}, k=1,2$$

where $Sample_{ik}$ stands for original biomarker measurement, $i$stands for batch, $X_{ik}$ is the median of QC sample in $i^{th}$ batch using $k^{th}$ QC sample, and$X_{.k}$ is the median from all batches using $k^{th}$QC sample. In this study, $QR_{k}$ equals to ratio between the median of 1st QC samples and the median of $k^{th}$ QC samples, and $QR_{1}$ equals to 1. Corrected biomarker measurement $Normalized_{ik}$was then used in all downstream analysis.

For comparison, model analyses were also conducted using raw assay results without correction.

1.5. **Statistical analysis: CV-based exclusion, potential selection bias, and sensitivity analysis**

Because batches with CVs exceeding 20% were excluded from the primary analysis, potential selection bias could be a concern. The OxS assays were initially developed and implemented in the SMHS samples, with subsequent methodological refinements leading to improved assay reliability in later batches from the SWHS and SCCS. Among the three OxS markers, 15‑F_2t_‑IsoP‑M consistently showed the lowest proportion of samples with CV >20%, followed by 15‑F_2t_‑IsoP and 5‑F_2t_‑IsoP. Given that 5‑F_2t_‑IsoP was the primary biomarker of the study, evaluation of CV‑based exclusions focused on this marker.

In the SWHS, only 1.75% of case–control pairs (22 of 1,255) had CVs >20% for 5‑F_2t_‑IsoP; and none of SCCS batches had CVs >20%. Thus, exclusions based on assay variability were minimal in these two cohorts and are unlikely to have introduced meaningful selection bias.

In the SMHS, a higher proportion of case–control pairs (20.2%, 138 of 683) had CVs >20% for 5‑F_2t_‑IsoP and were excluded from the primary analysis. However, comparison of baseline characteristics between participants with CV ≤20% and >20% showed that two groups were largely comparable, with standardized mean differences >0.20 observed only for ever smoking status (**Supplemental Tables 2-3**). No meaningful differences were observed for follow‑up time or other key demographic or lifestyle variables. In addition, sensitivity analyses conducted with and without these exclusions yielded materially similar results (**Tables 1-2** and **Supplemental Tables 12-13**).

These evaluations suggest that exclusion of samples with CV >20% is unlikely to have introduced substantial selection bias.

**Supplemental Table 1. Deviance residual diagnostic summary for urinary 5-F_2t_-IsoP by follow-up time**

| Time interval | N | Mean deviance residual | SD deviance residual | 95th percentile of \|residual\| | No. of \|residual\| >2 | % with \|residual\| >2 | Slope of deviance residual | P-value |
| --- | --- | --- | --- | --- | --- | --- | --- | --- |
| <5 years | 774 | -0.179 | 0.761 | 1.171 | 0 | 0 | -0.004 | 0.59 |
| 5-9 years | 935 | -0.183 | 0.780 | 1.147 | 0 | 0 | -0.002 | 0.77 |
| >9 years | 1844 | -0.188 | 0.802 | 1.079 | 0 | 0 | -0.002 | 0.80 |
| Overall | 3553 | -0.188 | 0.801 | 1.084 | 0 | 0 | -0.002 | 0.55 |

**Supplemental Table 2. Distributions of baseline characteristics and follow-up time by 5-F_2t_-IsoP CV categories in SMHS controls**

|  | CV < 20% (N=545) | CV ≥ 20% (N=138) | SMD ^a^ |
| --- | --- | --- | --- |
| Age at baseline (years), mean (SD) | 61.7 (9.0) | 61.8 (9.1) | 0.02 |
| Education, high school and above, n (%) | 306 (56.1%) | 73 (52.9%) | 0.07 |
| Ever smoking, n (%) | 360 (66.1%) | 77 (55.8%) | 0.21 |
| Ever alcohol dringking, n (%) | 193 (35.4%) | 45 (32.6%) | 0.06 |
| Body mass index (kg/m2), mean (SD) | 23.67 (2.9) | 24.1 (3.2) | 0.15 |
| Total energy intake (kcal/d), mean (SD) | 1875.0 (516.2) | 1972.1 (483.6) | 0.19 |
| Use of vitamin supplements, n (%) | 85 (15.6%) | 22 (15.9%) | 0.01 |
| Use of aspirin and other NSAIDs, n (%) | 51 (9.4%) | 14 (10.1%) | 0.03 |
| Family history of colorectal cancer, n (%) | 9 (1.7%) | 2 (1.4%) | 0.02 |
| Follow-up interval <5 years, n (%) | 200 (36.7%) | 41 (29.7%) | 0.15 |
| Follow-up interval 5-9 years, n (%) | 231 (42.4%) | 69 (50.0%) | 0.15 |
| Follow-up interval >9 years, n (%) | 114 (20.9%) | 28 (20.3%) | 0.02 |

^a^ SMD: standardized mean difference (Cohen’s d).

**Supplemental Table 3. Distributions of baseline characteristics and follow-up time by 5-F_2t_-IsoP CV categories in SMHS cases**

|  | CV < 20% (N=545) | CV ≥ 20% (N=138) | SMD ^a^ |
| --- | --- | --- | --- |
| Age at baseline (years), mean (SD) | 62.1 (9.1) | 62.0 (9.2) | 0.01 |
| Education, high school and above, n (%) | 290 (53.2%) | 83 (60.1%) | 0.14 |
| Ever smoking, n (%) | 342 (62.8%) | 99 (71.7%) | 0.19 |
| Ever alcohol dringking, n (%) | 181 (33.2%) | 58 (42.0%) | 0.18 |
| Body mass index (kg/m2), mean (SD) | 24.4 (3.2) | 24.12 (3.3) | 0.09 |
| Total energy intake (kcal/d), mean (SD) | 1896.2 (452.6) | 1880.7 (465.5) | 0.03 |
| Use of vitamin supplements, n (%) | 92 (16.9%) | 26 (18.8%) | 0.05 |
| Use of aspirin and other NSAIDs, n (%) | 73 (13.4%) | 20 (14.5%) | 0.03 |
| Family history of colorectal cancer, n (%) | 15 (2.8%) | 7 (5.1%) | 0.12 |
| Follow-up interval <5 years, n (%) | 200 (36.7%) | 41 (29.7%) | 0.15 |
| Follow-up interval 5-9 years, n (%) | 231 (42.4%) | 69 (50.0%) | 0.15 |
| Follow-up interval >9 years, n (%) | 114 (20.9%) | 28 (20.3%) | 0.02 |

^a^ SMD: standardized mean difference (Cohen’s d).

**Supplemental Table 4. Baseline characteristics of colorectal cancer cases and matched controls in the SWHS and the SMHS**

| **Characteristics** | **All** | | | | |  | **Females in SWHS** | | | | |  | **Males in SMHS** | | | | |
| --- | --- | --- | --- | --- | --- | --- | --- | --- | --- | --- | --- | --- | --- | --- | --- | --- | --- |
|  | **Cases** |  | **Controls** |  | ***P* value ^a^** |  | **Cases** |  | **Controls** |  | ***P* value ^a^** |  | **Cases** |  | **Controls** |  | ***P* value ^a^** |
|  | **(n = 1938)** |  | **(n = 1938)** |  |  |  | **(n = 1035)** |  | **(n = 1035)** |  |  |  | **(n = 903)** |  | **(n = 903)** |  |  |
| Age (year), mean (SD) | 59.4 (9.2) |  | 59.2 (9.2) |  | 0.741 |  | 57.5 (8.8) |  | 57.5 (8.9) |  | 0.981 |  | 61.5 (9.2) |  | 61.3 (9.2) |  | 0.630 |
| Education, high school and above, n (%) | 865 (44.6) |  | 844 (43.6) |  | 0.523 |  | 361 (34.9) |  | 342 (33.0) |  | 0.402 |  | 504 (55.8) |  | 502 (55.6) |  | 0.969 |
| Alcohol consumption, n (%) | 334 (17.2) |  | 346 (17.9) |  | 0.644 |  | 22 (2.1) |  | 26 (2.5) |  | 0.661 |  | 312 (34.6) |  | 320 (35.4) |  | 0.734 |
| Cigarette smoking, n (%) | 605 (31.2) |  | 628 (32.4) |  | 0.453 |  | 29 (2.8) |  | 38 (3.7) |  | 0.327 |  | 576 (63.8) |  | 590 (65.3) |  | 0.526 |
| Family history of colorectal cancer, n (%) | 65 (3.4) |  | 36 (1.9) |  | 0.0052 |  | 38 (3.7) |  | 19 (1.8) |  | 0.023 |  | 27 (3.0) |  | 17 (1.9) |  | 0.178 |
| Postmenopausal, n (%) | NA |  | NA |  | NA |  | 749 (72.4) |  | 759 (73.3) |  | 0.687 |  | NA |  | NA |  | NA |
| Physical activity (MET-h/wk), mean (SD) | 89.0 (44.0) |  | 88.8 (47.3) |  | 0.275 |  | 108.2 (41.5) |  | 109.7 (45.8) |  | 0.913 |  | 67.1 (35.7) |  | 64.7 (36.3) |  | 0.098 |
| Body mass index (kg/m^2^), mean (SD) | 24.4 (3.3) |  | 24.2 (3.4) |  | 0.069 |  | 24.5 (3.4) |  | 24.6 (3.6) |  | 0.549 |  | 24.3 (3.2) |  | 23.7 (3.0) |  | <0.001 |
| Use of vitamin supplements, n (%) | 348 (18.0) |  | 318 (16.4) |  | 0.228 |  | 189 (18.3) |  | 185 (17.9) |  | 0.864 |  | 159 (17.6) |  | 133 (14.7) |  | 0.112 |
| Use of aspirin and other NSAIDs, n (%) | 134 (6.9) |  | 115 (5.9) |  | 0.241 |  | 20 (1.9) |  | 31 (3.0) |  | 0.165 |  | 114 (12.6) |  | 84 (9.3) |  | 0.031 |
| Total energy intake (kcal/d), mean (SD) | 1775.6 (439.8) |  | 1786.4 (475.0) |  | 0.950 |  | 1673.5 (394.1) |  | 1693.9 (428.1) |  | 0.450 |  | 1892.6 (460.1) |  | 1892.5 (503.4) |  | 0.557 |
| Charlson comorbidity index, mean (SD) | 0.4 (0.9) |  | 0.4 (0.8) |  | 0.967 |  | 0.3 (0.7) |  | 0.3 (0.8) |  | 0.061 |  | 0.6 (1.0) |  | 0.5 (0.9) |  | 0.065 |

Abbreviations: SWHS, Shanghai Women's Health Study; SMHS, Shanghai Men's Health Study; case, study participant who had developed colorectal cancer during follow-up; control, study participant who remained cancer free; MET, metabolic equivalent; NSAID, nonsteroidal anti-inflammatory drug; NA, not applicable.

^a^ Wilcoxon rank sum test was used for continuous variables and chi-square test for categorical variables.

**Supplemental Table 5. Baseline characteristics of colorectal cancer cases and matched controls in the SCCS**

| **Characteristics** | **All** | | | | |  | **African Americans** | | | | |  | **European Americans** | | | | |
| --- | --- | --- | --- | --- | --- | --- | --- | --- | --- | --- | --- | --- | --- | --- | --- | --- | --- |
|  | **Cases** |  | **Controls** |  | ***P* value ^a^** |  | **Cases** |  | **Controls** |  | ***P* value ^a^** |  | **Cases** |  | **Controls** |  | ***P* value ^a^** |
|  | **(n = 285)** |  | **(n = 570)** |  |  |  | **(n = 192)** |  | **(n = 384)** |  |  |  | **(n = 93)** |  | **(n = 186)** |  |  |
| Age (year), mean (SD) | 55.1 (8.8) |  | 55.0 (8.7) |  | 0.846 |  | 54.6 (8.8) |  | 54.4 (8.7) |  | 0.793 |  | 56.1 (8.7) |  | 56.1 (8.6) |  | 0.982 |
| Education, high school and above, n (%) | 181 (63.5) |  | 359 (63.0) |  | 0.895 |  | 115 (59.9) |  | 231 (60.2) |  | 0.995 |  | 66 (71.0) |  | 128 (68.8) |  | 0.821 |
| Alcohol consumption, n (%) | 237 (83.2) |  | 478 (83.9) |  | 0.997 |  | 165 (85.9) |  | 325 (84.6) |  | 0.827 |  | 72 (77.4) |  | 153 (82.3) |  | 0.646 |
| Cigarette smoking, n (%) | 180 (63.2) |  | 371 (65.1) |  | 0.736 |  | 116 (60.4) |  | 243 (63.3) |  | 0.629 |  | 64 (68.8) |  | 128 (68.8) |  | 0.998 |
| Family history of colorectal cancer, n (%) | 34 (11.9) |  | 43 (7.5) |  | 0.044 |  | 18 (9.4) |  | 26 (6.8) |  | 0.345 |  | 16 (17.2) |  | 17 (9.1) |  | 0.074 |
| Gender, male (%) | 112 (39.3) |  | 224 (39.3) |  | 0.993 |  | 80 (41.7) |  | 160 (41.7) |  | 0.994 |  | 32 (34.4) |  | 64 (34.4) |  | 0.992 |
| Physical activity (MET-h/wk), mean (SD) | 160.0 (136.5) |  | 160.7 (140.5) |  | 0.909 |  | 156.5 (142.4) |  | 152.9 (141.8) |  | 0.715 |  | 167.4 (123.6) |  | 176.8 (136.9) |  | 0.705 |
| Body mass index (kg/m^2^), mean (SD) | 30.7 (7.9) |  | 31.2 (7.6) |  | 0.234 |  | 30.1 (7.4) |  | 31.2 (8.0) |  | 0.142 |  | 31.8 (8.7) |  | 31.1 (6.9) |  | 0.873 |
| Use of vitamin supplements, n (%) | 116 (40.7) |  | 249 (43.7) |  | 0.491 |  | 78 (40.6) |  | 156 (40.6) |  | 0.990 |  | 38 (40.9) |  | 93 (50.0) |  | 0.258 |
| Use of aspirin and other NSAIDs, n (%) | 32 (11.2) |  | 73 (12.8) |  | 0.590 |  | 21 (10.9) |  | 50 (13.0) |  | 0.567 |  | 11 (11.8) |  | 23 (12.4) |  | 0.995 |
| Total energy intake (kcal/d), mean (SD) | 2596.9 (1540.5) |  | 2547.2 (1439.6) |  | 0.892 |  | 2726.8 (1591.5) |  | 2692.5 (1524.6) |  | 0.871 |  | 2334.3 (1403.7) |  | 2262.3 (1210.5) |  | 0.863 |
| Charlson comorbidity index, mean (SD) | 2.0 (1.5) |  | 2.1 (1.4) |  | 0.309 |  | 1.9 (1.5) |  | 2.1 (1.4) |  | 0.108 |  | 2.3 (1.6) |  | 2.2 (1.4) |  | 0.540 |

Abbreviations: SCCS, Southern Community Cohort Study; case, study participant who had developed colorectal cancer; control, study participant who remained cancer free; MET, metabolic equivalent; NSAID, nonsteroidal anti-inflammatory drug.

^a^ Wilcoxon rank sum test was used for continuous variables and chi-square test for categorical variables

**Supplemental Table 6. Urinary F_2_-IsoP levels (ng/mg Cr) in colorectal cancer cases and controls from the SWHS, SMHS and SCCS**

|  | **Cases** | |  | **Controls** | |  | **Difference b/w case and control ^a^** | ***P* value ^b^** |
| --- | --- | --- | --- | --- | --- | --- | --- | --- |
|  | **Geometric mean** | **95% CI** |  | **Geometric mean** | **95% CI** |  |  |  |
| **SWHS and SMHS** | | | | | | | | |
| 5-F_2t_-IsoP | 6.22 | (6.02, 6.43) |  | 6.76 | (6.54, 6.99) |  | -8.0% | < 0.001 |
| 15-F2t-IsoP | 2.13 | (2.06, 2.21) |  | 2.16 | (2.09, 2.24) |  | -1.4% | 0.602 |
| 15-F_2t_-IsoP-M | 9.07 | (8.73, 9.42) |  | 9.73 | (9.37, 10.10) |  | -6.8% | 0.007 |
| RPS-IsoP | 556% | (534%, 578%) |  | 588% | (565%, 612%) |  | -5.4% | 0.043 |
| **SCCS** | | | | | | | | |
| 5-F_2t_-IsoP | 7.27 | (6.58, 8.03) |  | 7.93 | (7.39, 8.52) |  | -8.3% | 0.165 |
| 15-F2t-IsoP | 1.67 | (1.54, 1.82) |  | 1.75 | (1.65, 1.86) |  | -4.6% | 0.361 |
| 15-F_2t_-IsoP-M | 14.09 | (12.83, 15.49) |  | 14.76 | (13.80, 15.78) |  | -4.5% | 0.440 |
| RPS-IsoP | 1320% | (1200%, 1453%) |  | 1318% | (1231%, 1410%) |  | 0.2% | 0.974 |

Abbreviations: 5-F_2t_-IsoP, 5-F_2t_-Isoprostane; 15-F_2t_-IsoP, 15-F_2t_-Isoprostane; 15-F_2t_-IsoP-M, 2,3-Dinor-5,6-dihydro-15-F_2t_-Isoprostane; RPS-IsoP, ratio of 15-F_2t_-IsoP-M (β-oxidation product) to 15-F_2t_-IsoP (substrate); SWHS, Shanghai Women's Health Study; SMHS, Shanghai Men's Health Study; SCCS, Southern Community Cohort Study; All biomarker values are centered by batch median.

Total sizes of case: control in the SWHS and SMHS: 1938:1938; in the SCCS: 285: 570.

^a^ Difference (%) = (geometric mean of biomarker in case – geometric mean of biomarker in control)/geometric mean of biomarker in control.

^b^ Derived from paired *t-*tests for SWHS and SMHS and from mixed-effects models for SCCS due to 1:2 matching.

**Supplemental Table 7. Associations between baseline urinary F_2_-IsoP levels and subsequent colorectal cancer risk by sex in the SWHS and SMHS**

|  | **Number of pairs ^c^** | **OR (95% CI) for colorectal cancer risk relative to reference, ^a^ by percentile distribution of biomarkers ^b^** | | | | | ***P* for overall association** | ***P* for nonlinear association** |  |
| --- | --- | --- | --- | --- | --- | --- | --- | --- | --- |
|  |  | **10th** | **30th** | **50th** | **70th** | **90th** |  |  |  |
| **In the SWHS (Females)** | | | | | | | | | |
| 5-F_2t_-IsoP | 1013 | 1.07 (0.99, 1.17) | 1.04 (0.99, 1.10) | Reference | 0.92 (0.84, 1.02) | 0.78 (0.57, 1.06) | 0.262 | 0.518 |  |
| 15-F2t-IsoP | 1013 | 0.98 (0.93, 1.03) | 1.00 (0.99, 1.02) | Reference | 0.96 (0.91, 1.01) | 0.85 (0.71, 1.02) | 0.100 | 0.050 |  |
| 15-F_2t_-IsoP-M | 1035 | 1.11 (0.99, 1.24) | 1.06 (0.99, 1.13) | Reference | 0.92 (0.83, 1.02) | 0.80 (0.60, 1.06) | 0.031 | 0.657 |  |
| RPS-IsoP | 1013 | 1.19 (0.96, 1.48) | 1.08 (0.99, 1.18) | Reference | 0.93 (0.86, 0.99) | 0.83 (0.72, 0.95) | 0.027 | 0.398 |  |
| **In the SMHS (Males)** | | | | | | | | | |
| 5-F2t-IsoP | 765 | 1.27 (1.11, 1.46) | 1.12 (1.05, 1.19) | Reference | 0.88 (0.82, 0.94) | 0.80 (0.70, 0.92) | 0.001 | 0.009 |  |
| 15-F2t-IsoP | 604 | 1.06 (0.97, 1.17) | 1.02 (0.99, 1.05) | Reference | 0.96 (0.90, 1.02) | 0.95 (0.86, 1.07) | 0.409 | 0.196 |  |
| 15-F2t-IsoP-M | 884 | 1.15 (1.05, 1.26) | 1.07 (1.02, 1.11) | Reference | 0.94 (0.89, 1.00) | 0.86 (0.71, 1.05) | 0.005 | 0.179 |  |
| RPS-IsoP | 586 | 1.14 (0.93, 1.40) | 1.05 (0.97, 1.14) | Reference | 0.96 (0.90, 1.03) | 0.94 (0.85, 1.04) | 0.302 | 0.203 |  |

Abbreviations: 5-F_2t_-IsoP, 5-F_2t_-Isoprostane; 15-F_2t_-IsoP, 15-F_2t_-Isoprostane; 15-F_2t_-IsoP-M, 2,3-Dinor-5,6-dihydro-15-F_2t_-Isoprostane; RPS-IsoP, ratio of 15-F_2t_-IsoP-M (β-oxidation product) to 15-F_2t_-IsoP (substrate); CI, confidence interval; ng/mg Cr, nanograms per milligram of creatinine; OR, odds ratio; SMHS, Shanghai Men's Health Study; SWHS, Shanghai Women's Health Study.

^a^ Multivariable ORs estimated using a conditional logistic regression model with restricted cubic-spline functions, using the 50th percentile of biomarkers in controls as the reference; and adjusted for age at baseline, education, cigarette smoking, alcohol consumption, BMI, physical activity, menopausal status (for female participants), regular use of vitamin supplements, regular use of aspirin and other nonsteroidal anti-inflammatory drugs, Charlson comorbidity score, family history of colorectal cancer in first-degree relatives, and total energy intake.

^b^ Cutoffs were based on the percentile distribution of levels in controls— for 5-F_2t_-IsoP (ng/mg Cr): 1.00 (10th), 1.56 (30th), 2.02 (50th), 2.69 (70th), and 4.28 (90th) in the SWHS; and 1.81 (10th), 2.97 (30th), 4.41 (50th), 6.23 (70th) and 9.64 (90th) in the SMHS; for 15-F_2t_-IsoP (ng/mg Cr): 0.59 (10th), 0.91 (30th), 1.00 (50th), 1.34 (70th), and 2.26 (90th) in the SWHS, and 0.86 (10th), 1.07 (30th), 1.75 (50th), 2.65 (70th) and 4.58 (90th) in the SMHS. for 15-F_2t_-IsoP-M (ng/mg Cr): 4.46 (10th), 6.53 (30th), 8.83 (50th), 12.83 (70th), and 21.88 (90th) in the SWHS; and 4.41 (10th), 9.12 (30th), 13.21 (50th), 18.48 (70th) and 28.79 (90th) in the SMHS; for RPS-IsoP: 422.7% (10th), 649.7% (30th), 853.0% (50th), 1113.5% (70th), and 1770.1% (90th) in the SWHS, and 233.1% (10th), 465.6% (30th), 770.1% (50th), 1180.8% (70th) and 1955.3% (90th) in the SMHS.

^c^ The number of case-control pairs in the analysis after exclusion of batches with CV >20%, with the case-control ratio of 1:1.

**Supplemental Table 8. Associations between baseline urinary F_2_-IsoP levels and subsequent colorectal cancer risk by race in the SCCS**

|  | **Number of pairs ^c^** | **OR (95% CI) for colorectal cancer risk relative to reference, ^a^ by percentile distribution of biomarkers ^b^** | | | | | ***P* for overall association** | ***P* for nonlinear association** |
| --- | --- | --- | --- | --- | --- | --- | --- | --- |
|  |  | **10th** | **30th** | **50th** | **70th** | **90th** |  |  |
| **African Americans** | | | | | | | |  |
| 5-F_2t_-IsoP | 192 | 1.17 (0.93, 1.47) | 1.09 (0.96, 1.23) | Reference | 0.95 (0.88, 1.03) | 0.97 (0.76, 1.24) | 0.397 | 0.212 |
| 15-F_2t_-IsoP | 192 | 1.05 (0.94, 1.18) | 1.01 (0.99, 1.04) | Reference | 0.98 (0.94, 1.01) | 0.92 (0.80, 1.07) | 0.466 | 0.657 |
| 15-F_2t_-IsoP-M | 192 | 1.13 (1.00, 1.28) | 1.03 (0.98, 1.08) | Reference | 1.01 (0.94, 1.09) | 1.05 (0.82, 1.35) | 0.064 | 0.029 |
| RPS-IsoP | 192 | 1.15 (0.76, 1.75) | 1.07 (0.91, 1.26) | Reference | 0.92 (0.82, 1.03) | 0.78 (0.57, 1.07) | 0.275 | 0.923 |
| **European Americans** | | | | | | | | |
| 5-F_2t_-IsoP | 92 | 1.33 (0.99, 1.78) | 1.18 (0.99, 1.40) | Reference | 0.86 (0.72, 1.03) | 0.91 (0.66, 1.25) | 0.075 | 0.030 |
| 15-F_2t_-IsoP | 92 | 1.03 (0.81, 1.32) | 1.01 (0.94, 1.08) | Reference | 0.99 (0.89, 1.10) | 1.01 (0.80, 1.28) | 0.924 | 0.740 |
| 15-F_2t_-IsoP-M | 92 | 0.91 (0.68, 1.21) | 0.96 (0.86, 1.07) | Reference | 1.04 (0.93, 1.17) | 1.11 (0.75, 1.64) | 0.739 | 0.754 |
| RPS-IsoP | 92 | 0.74 (0.39, 1.43) | 0.88 (0.69, 1.14) | Reference | 1.11 (0.94, 1.30) | 1.23 (0.83, 1.83) | 0.461 | 0.630 |

Abbreviations: 5-F_2t_-IsoP, 5-F_2t_-Isoprostane; 15-F_2t_-IsoP, 15-F_2t_-Isoprostane; 15-F_2t_-IsoP-M, 2,3-Dinor-5,6-dihydro-15-F_2t_-Isoprostane; RPS-IsoP, ratio of 15-F_2t_-IsoP-M (metabolic product) to 15-F_2t_-IsoP (substrate); CI, confidence interval; ng/mg Cr, nanograms per milligram of creatinine; OR, odds ratio; SCCS, Southern Community Cohort Study.

^a^ Multivariable ORs estimated using a conditional logistic regression model with restricted cubic-spline functions, using the 50th percentile of biomarkers in controls as the reference; and adjusted for age at baseline, education, cigarette smoking, alcohol consumption, BMI, physical activity, menopausal status (for female participants), regular use of vitamin supplements, regular use of aspirin and other nonsteroidal anti-inflammatory drugs, Charlson comorbidity score, family history of colorectal cancer in first-degree relatives, and total energy intake.

^b^ Cutoffs were based on the percentile distribution of biomarker levels in controls— for 5-F_2t_-IsoP (ng/mg Cr): 0.69 (10th), 1.07 (30th), 1.70 (50th), 2.73 (70th), and 5.05 (90th) in African Americans in SCCS; and 0.94 (10th), 1.61 (30th), 2.42 (50th), 4.04 (70th) and 6.78 (90th) in European Americans in SCCS; for 15-F_2t_-IsoP (ng/mg Cr): 0.59 (10th), 0.91 (30th), 1.00 (50th), 1.34 (70th), and 2.26 (90th) in African Americans in SCCS, and 0.57 (10th), 1.00 (30th), 1.29 (50th), 1.78 (70th) and 3.08 (90th) in European Americans in SCCS. for 15-F_2t_-IsoP-M (ng/mg Cr): 6.33 (10th), 10.48 (30th), 13.94 (50th), 19.09 (70th), and 29.15 (90th) in African Americans in SCCS; and 7.06 (10th), 11.34 (30th), 16.75 (50th), 24.17 (70th) and 35.27 (90th) in European Americans in SCCS; for RPS-IsoP: 517.8% (10th), 982.6% (30th), 1412.6% (50th), 1796.7% (70th), and 2625.8% (90th) in African Americans in SCCS, and 472.5% (10th), 922.3% (30th), 1252.5% (50th), 1789.6% (70th) and 2522.7% (90th) in European Americans in SCCS.

^c^ The number of case-control pairs with the case-control ratio of 1:2. All intra-batch CVs were <20%.

**Supplemental Table 9. Associations between baseline urinary F_2_-IsoP levels and subsequent colorectal cancer risk by time interval between urine sample collection at baseline and cancer diagnosis during follow-up in the SWHS**

|  | **Number of pairs ^c^** | **OR (95% CI) for colorectal cancer risk relative to reference, ^a^ by percentile distribution of biomarkers ^b^** | | | | | ***P* for overall association** | ***P* for nonlinear association** | |  |
| --- | --- | --- | --- | --- | --- | --- | --- | --- | --- | --- |
|  |  | **10th** | **30th** | **50th** | **70th** | **90th** |  |  |  |  |
| **Cases diagnosed <5 years after enrollment** | | | | | | | | |  | |
| 5-F_2t_-IsoP | 184 | 1.27 (1.03, 1.56) | 1.13 (1.01, 1.26) | Reference | 0.83 (0.71, 0.99) | 0.52 (0.29, 0.95) | 0.081 | 0.435 | |  |
| 15-F_2t_-IsoP | 184 | 1.01 (0.92, 1.12) | 1.00 (0.98, 1.02) | Reference | 1.01 (0.93, 1.09) | 1.04 (0.76, 1.42) | 0.886 | 0.622 | |  |
| 15-F_2t_-IsoP-M | 185 | 1.45 (0.99, 2.12) | 1.22 (0.99, 1.50) | Reference | 0.71 (0.51, 1.01) | 0.32 (0.10, 1.03) | 0.135 | 0.369 | |  |
| RPS-IsoP | 184 | 0.87 (0.54, 1.41) | 0.97 (0.81, 1.16) | Reference | 0.95 (0.83, 1.08) | 0.63 (0.35, 1.14) | 0.275 | 0.244 | |  |
| **Cases diagnosed 5-9 years after enrollment** | | | | | | | | |  | |
| 5-F_2t_-IsoP | 214 | 1.12 (0.90, 1.39) | 1.09 (0.97, 1.22) | Reference | 0.86 (0.71, 1.04) | 0.58 (0.30, 1.16) | 0.115 | 0.038 | |  |
| 15-F_2t_-IsoP | 214 | 0.95 (0.87, 1.03) | 1.00 (0.99, 1.01) | Reference | 0.98 (0.94, 1.03) | 0.91 (0.76, 1.10) | 0.197 | 0.075 | |  |
| 15-F_2t_-IsoP-M | 216 | 1.04 (0.91, 1.19) | 1.02 (0.95, 1.10) | Reference | 0.97 (0.86, 1.09) | 0.89 (0.59, 1.33) | 0.376 | 0.391 | |  |
| RPS-IsoP | 214 | 1.55 (1.04, 2.29) | 1.18 (1.01, 1.37) | Reference | 0.94 (0.85, 1.04) | 1.16 (0.75, 1.80) | 0.081 | 0.027 | |  |
| **Cases diagnosed >9 years after enrollment** | | | | | | | | |  | |
| 5-F_2t_-IsoP | 615 | 1.02 (0.96, 1.08) | 1.00 (0.98, 1.03) | Reference | 1.00 (0.95, 1.05) | 1.00 (0.84, 1.18) | 0.746 | 0.543 | |  |
| 15-F_2t_-IsoP | 615 | 0.96 (0.89, 1.03) | 1.00 (0.99, 1.01) | Reference | 1.00 (0.97, 1.03) | 0.94 (0.83, 1.06) | 0.248 | 0.110 | |  |
| 15-F_2t_-IsoP-M | 614 | 1.06 (0.97, 1.17) | 1.03 (0.98, 1.09) | Reference | 0.95 (0.86, 1.05) | 0.84 (0.59, 1.20) | 0.204 | 0.764 | |  |
| RPS-IsoP | 614 | 1.14 (0.93, 1.41) | 1.06 (0.97, 1.17) | Reference | 0.94 (0.87, 1.01) | 0.83 (0.70, 0.98) | 0.089 | 0.545 | |  |

Abbreviations: 5-F_2t_-IsoP, 5-F_2t_-Isoprostane; 15-F_2t_-IsoP, 15-F_2t_-Isoprostane; 15-F_2t_-IsoP-M, 2,3-Dinor-5,6-dihydro-15-F_2t_-Isoprostane; RPS-IsoP, ratio of 15-F_2t_-IsoP-M (β-oxidation product) to 15-F_2t_-IsoP (substrate); CI, confidence interval; ng/mg Cr, nanograms per milligram of creatinine; OR, odds ratio; SWHS, Shanghai Women's Health Study.

^a^ Multivariable ORs estimated using a conditional logistic regression model with restricted cubic-spline functions, stratified by the time interval between the baseline sample collection and cancer diagnosis during follow-up, using the 50th percentile of biomarkers in controls as the reference; and multivariable adjusted for age at baseline, education, cigarette smoking, alcohol consumption, BMI, physical activity, menopausal status, regular use of vitamin supplements, regular use of aspirin and other nonsteroidal anti-inflammatory drugs, Charlson comorbidity score, family history of colorectal cancer in first-degree relatives, and total energy intake.

^b^ Cutoffs were based on the percentile distribution of levels in controls— for 5-F_2t_-IsoP (ng/mg Cr): 1.00 (10th), 1.56 (30th), 2.02 (50th), 2.69 (70th), and 4.28 (90th) in the SWHS. for 15-F_2t_-IsoP (ng/mg Cr): 0.59 (10th), 0.91 (30th), 1.00 (50th), 1.34 (70th), and 2.26 (90th) in the SWHS. for 15-F_2t_-IsoP-M (ng/mg Cr): 4.46 (10th), 6.53 (30th), 8.83 (50th), 12.83 (70th), and 21.88 (90th) in the SWHS. for RPS-IsoP: 422.7% (10th), 649.7% (30th), 853.0% (50th), 1113.5% (70th), and 1770.1% (90th) in the SWHS.

^c^ The number of case-control pairs after exclusion of batches with CV >20%.

**Supplemental Table 10. Associations between baseline urinary F_2_-IsoP levels and subsequent colorectal cancer risk by time interval between urine sample collection at baseline and cancer diagnosis during follow-up in the SMHS**

|  | **Number of pairs ^c^** | **OR (95% CI) for colorectal cancer risk relative to reference, ^a^** **by percentile distribution of biomarkers ^b^** | | | | | ***P* for overall association** | | ***P* for nonlinear association** | |
| --- | --- | --- | --- | --- | --- | --- | --- | --- | --- | --- |
|  |  | **10th** | **30th** | **50th** | **70th** | **90th** |  |  |  |  |
| **Cases diagnosed <5 years after enrollment** | | | | | | | |  | |  |
| 5-F_2t_-IsoP | 203 | 2.14 (1.44, 3.18) | 1.38 (1.18, 1.61) | Reference | 0.83 (0.72, 0.96) | 0.73 (0.43, 1.24) | < 0.001 | | 0.017 | |
| 15-F_2t_-IsoP | 183 | 1.12 (0.90, 1.39) | 1.08 (0.93, 1.26) | Reference | 0.96 (0.87, 1.05) | 0.95 (0.77, 1.16) | 0.601 | | 0.364 | |
| 15-F_2t_-IsoP-M | 226 | 1.10 (0.88, 1.37) | 1.01 (0.92, 1.11) | Reference | 1.02 (0.88, 1.19) | 1.07 (0.69, 1.67) | 0.490 | | 0.283 | |
| RPS-IsoP | 165 | 1.10 (0.73, 1.66) | 1.05 (0.85, 1.29) | Reference | 0.95 (0.74, 1.20) | 0.91 (0.59, 1.40) | 0.635 | | 0.628 | |
| **Cases diagnosed 5-9 years after enrollment** | | | | | | | |  | |  |
| 5-F_2t_-IsoP | 255 | 1.52 (1.15, 2.02) | 1.19 (1.06, 1.34) | Reference | 0.91 (0.85, 0.98) | 0.91 (0.75, 1.11) | 0.011 | | 0.011 | |
| 15-F_2t_-IsoP | 144 | 1.40 (1.07, 1.83) | 1.26 (1.05, 1.53) | Reference | 0.90 (0.81, 1.00) | 0.96 (0.73, 1.26) | 0.053 | | 0.021 | |
| 15-F_2t_-IsoP-M | 323 | 1.18 (1.00, 1.41) | 1.07 (1.00, 1.14) | Reference | 0.93 (0.84, 1.03) | 0.81 (0.60, 1.10) | 0.101 | | 0.711 | |
| RPS-IsoP | 144 | 0.88 (0.54, 1.44) | 0.94 (0.73, 1.20) | Reference | 1.08 (0.80, 1.47) | 1.15 (0.68, 1.95) | 0.832 | | 0.619 | |
| **Cases diagnosed >9 years after enrollment** | | | | | | | |  | |  |
| 5-F_2t_-IsoP | 307 | 1.09 (0.89, 1.33) | 1.08 (0.97, 1.20) | Reference | 0.87 (0.73, 1.04) | 0.64 (0.37, 1.12) | 0.291 | | 0.309 | |
| 15-F_2t_-IsoP | 277 | 0.96 (0.84, 1.11) | 0.98 (0.89, 1.07) | Reference | 0.99 (0.90, 1.10) | 0.98 (0.72, 1.34) | 0.851 | | 0.581 | |
| 15-F_2t_-IsoP-M | 335 | 1.35 (1.06, 1.71) | 1.12 (1.02, 1.23) | Reference | 0.89 (0.76, 1.03) | 0.72 (0.47, 1.12) | 0.035 | | 0.534 | |
| RPS-IsoP | 277 | 1.45 (0.95, 2.23) | 1.22 (0.97, 1.53) | Reference | 0.75 (0.53, 1.04) | 0.64 (0.37, 1.11) | 0.166 | | 0.070 | |

Abbreviations: 5-F_2t_-IsoP, 5-F_2t_-Isoprostane; 15-F_2t_-IsoP, 15-F_2t_-Isoprostane; 15-F_2t_-IsoP-M, 2,3-Dinor-5,6-dihydro-15-F_2t_-Isoprostane; RPS-IsoP, ratio of 15-F_2t_-IsoP-M (β-oxidation product) to 15-F_2t_-IsoP (substrate); CI, confidence interval; ng/mg Cr, nanograms per milligram of creatinine; OR, odds ratio; SMHS, Shanghai Men's Health Study.

^a^ Multivariable ORs estimated using a conditional logistic regression model with restricted cubic-spline functions, stratified by the time interval between the baseline sample collection and cancer diagnosis during follow-up, using the 50th percentile of biomarkers in controls as the reference; and multivariable adjusted for age at baseline, education, cigarette smoking, alcohol consumption, BMI, physical activity, regular use of vitamin supplements, regular use of aspirin and other nonsteroidal anti-inflammatory drugs, Charlson comorbidity score, family history of colorectal cancer in first-degree relatives, and total energy intake.

^b^ Cutoffs were based on the percentile distribution of levels in controls— for 5-F_2t_-IsoP (ng/mg Cr): 1.81 (10th), 2.97 (30th), 4.41 (50th), 6.23 (70th) and 9.64 (90th) in the SMHS; for 15-F_2t_-IsoP (ng/mg Cr): 0.86 (10th), 1.07 (30th), 1.75 (50th), 2.65 (70th) and 4.58 (90th) in the SMHS. for 15-F_2t_-IsoP-M (ng/mg Cr): 4.41 (10th), 9.12 (30th), 13.21 (50th), 18.48 (70th) and 28.79 (90th) in the SMHS; for RPS-IsoP: 233.1% (10th), 465.6% (30th), 770.1% (50th), 1180.8% (70th) and 1955.3% (90th) in the SMHS.

^c^ The number of case-control pairs after exclusion of batches with CV > 20%.

**Supplemental Table 11. Associations between baseline urinary F_2_-IsoP levels and subsequent colorectal cancer risk in the SWHS and the SMHS, stratified by anatomical sites and time interval between urine sample collection at baseline and cancer diagnosis during follow-up**

|  | **Number of pairs ^c^** | **OR (95% CI) for colorectal cancer risk relative to reference, ^a^ by percentile distribution of biomarkers ^b^** | | | | | ***P* for overall association** | ***P* for nonlinear association** |
| --- | --- | --- | --- | --- | --- | --- | --- | --- |
|  |  | **10^th^** | **30th** | **50th** | **70th** | **90th** |  |  |
| **Overall by anatomic site** |  |  |  |  |  |  |  |  |
| 5-F_2t_-IsoP |  |  |  |  |  |  |  |  |
| Colon cancer | 1127 | 1.14 (1.03, 1.25) | 1.06 (1.02, 1.10) | Reference | 0.92 (0.86, 0.97) | 0.77 (0.61, 0.97) | 0.004 | 0.432 |
| Rectal cancer | 615 | 1.10 (0.96, 1.26) | 1.05 (0.99, 1.11) | Reference | 0.95 (0.90, 1.00) | 0.88 (0.76, 1.02) | 0.121 | 0.422 |
| 15-F_2t_-IsoP |  |  |  |  |  |  |  |  |
| Colon cancer | 1031 | 1.00 (0.94, 1.07) | 1.00 (0.99, 1.02) | Reference | 0.98 (0.95, 1.01) | 0.91 (0.81, 1.02) | 0.287 | 0.618 |
| Rectal cancer | 586 | 0.97 (0.87, 1.07) | 0.99 (0.97, 1.02) | Reference | 1.01 (0.96, 1.05) | 0.97 (0.87, 1.08) | 0.575 | 0.400 |
| 15-F_2t_-IsoP-M |  |  |  |  |  |  |  |  |
| Colon cancer | 1219 | 1.10 (1.02, 1.18) | 1.04 (1.00, 1.08) | Reference | 0.96 (0.90, 1.03) | 0.90 (0.74, 1.10) | 0.007 | 0.103 |
| Rectal cancer | 700 | 1.12 (1.04, 1.21) | 1.07 (1.02, 1.12) | Reference | 0.90 (0.82, 0.98) | 0.73 (0.55, 0.96) | 0.017 | 0.967 |
| RPS-IsoP |  |  |  |  |  |  |  |  |
| Colon cancer | 1023 | 1.28 (1.04, 1.56) | 1.11 (1.02, 1.21) | Reference | 0.92 (0.85, 0.98) | 0.87 (0.77, 0.98) | 0.059 | 0.018 |
| Rectal cancer | 604 | 1.09 (0.84, 1.41) | 1.03 (0.93, 1.15) | Reference | 0.97 (0.87, 1.07) | 0.96 (0.82, 1.12) | 0.548 | 0.501 |
| **Colon cancer by time internal** | | | | | | | |  |
| 5-F_2t_-IsoP |  |  |  |  |  |  |  |  |
| Cases diagnosed <5 years | 228 | 1.67 (1.24, 2.25) | 1.25 (1.10, 1.41) | Reference | 0.79 (0.68, 0.91) | 0.64 (0.38, 1.08) | 0.001 | 0.024 |
| Cases diagnosed 5-9 years | 290 | 1.17 (0.95, 1.44) | 1.08 (0.98, 1.17) | Reference | 0.90 (0.82, 1.00) | 0.77 (0.54, 1.10) | 0.129 | 0.546 |
| Cases diagnosed >9 years | 609 | 1.05 (0.95, 1.16) | 1.02 (0.97, 1.07) | Reference | 0.98 (0.88, 1.08) | 0.92 (0.64, 1.33) | 0.674 | 0.713 |
| 15-F_2t_-IsoP |  |  |  |  |  |  |  |  |
| Cases diagnosed <5 years | 216 | 1.04 (0.91, 1.20) | 1.01 (0.97, 1.06) | Reference | 0.97 (0.89, 1.06) | 0.97 (0.81, 1.16) | 0.839 | 0.580 |
| Cases diagnosed 5-9 years | 222 | 0.96 (0.82, 1.13) | 1.00 (0.95, 1.04) | Reference | 0.95 (0.86, 1.05) | 0.71 (0.48, 1.04) | 0.199 | 0.228 |
| Cases diagnosed >9 years | 593 | 1.01 (0.92, 1.10) | 1.00 (0.98, 1.03) | Reference | 0.98 (0.93, 1.03) | 0.92 (0.77, 1.10) | 0.657 | 0.825 |
| 15-F_2t_-IsoP-M |  |  |  |  |  |  |  |  |
| Cases diagnosed <5 years | 243 | 1.14 (0.96, 1.34) | 1.02 (0.94, 1.11) | Reference | 1.02 (0.88, 1.19) | 1.07 (0.71, 1.63) | 0.031 | 0.021 |
| Cases diagnosed 5-9 years | 334 | 1.08 (0.95, 1.23) | 1.03 (0.96, 1.11) | Reference | 0.96 (0.86, 1.08) | 0.89 (0.65, 1.22) | 0.327 | 0.484 |
| Cases diagnosed >9 years | 642 | 1.10 (0.97, 1.24) | 1.04 (0.97, 1.12) | Reference | 0.95 (0.83, 1.07) | 0.86 (0.61, 1.23) | 0.179 | 0.599 |
| RPS-IsoP |  |  |  |  |  |  |  |  |
| Cases diagnosed <5 years | 208 | 1.32 (0.82, 2.12) | 1.12 (0.92, 1.36) | Reference | 0.92 (0.79, 1.06) | 0.87 (0.69, 1.09) | 0.416 | 0.259 |
| Cases diagnosed 5-9 years | 222 | 1.50 (0.95, 2.37) | 1.19 (0.98, 1.43) | Reference | 0.88 (0.76, 1.02) | 0.82 (0.66, 1.04) | 0.185 | 0.075 |
| Cases diagnosed >9 years | 593 | 1.23 (0.94, 1.60) | 1.10 (0.97, 1.24) | Reference | 0.92 (0.83, 1.03) | 0.89 (0.74, 1.07) | 0.245 | 0.108 |
| **Rectal cancer by time internal** | | | | | | | |  |
| 5-F_2t_-IsoP |  |  |  |  |  |  |  |  |
| Cases diagnosed <5 years | 159 | 1.42 (0.98, 2.04) | 1.17 (1.01, 1.37) | Reference | 0.83 (0.70, 0.99) | 0.66 (0.36, 1.21) | 0.078 | 0.338 |
| Cases diagnosed 5-9 years | 179 | 1.10 (0.82, 1.47) | 1.04 (0.92, 1.18) | Reference | 0.95 (0.86, 1.06) | 0.91 (0.75, 1.09) | 0.577 | 0.670 |
| Cases diagnosed >9 years | 313 | 1.06 (0.90, 1.25) | 1.03 (0.97, 1.11) | Reference | 0.94 (0.83, 1.05) | 0.82 (0.55, 1.20) | 0.519 | 0.923 |
| 15-F_2t_-IsoP |  |  |  |  |  |  |  |  |
| Cases diagnosed <5 years | 151 | 1.19 (0.95, 1.47) | 1.04 (0.98, 1.11) | Reference | 0.96 (0.84, 1.09) | 1.15 (0.75, 1.78) | 0.213 | 0.079 |
| Cases diagnosed 5-9 years | 136 | 1.04 (0.84, 1.30) | 1.01 (0.95, 1.08) | Reference | 0.97 (0.86, 1.08) | 0.92 (0.78, 1.08) | 0.500 | 0.819 |
| Cases diagnosed >9 years | 299 | 0.87 (0.76, 1.00) | 0.98 (0.95, 1.01) | Reference | 0.97 (0.89, 1.07) | 0.84 (0.58, 1.21) | 0.078 | 0.024 |
| 15-F_2t_-IsoP-M |  |  |  |  |  |  |  |  |
| Cases diagnosed <5 years | 168 | 0.98 (0.76, 1.27) | 1.00 (0.85, 1.17) | Reference | 1.00 (0.75, 1.34) | 1.00 (0.42, 2.35) | 0.921 | 0.764 |
| Cases diagnosed 5-9 years | 210 | 1.19 (1.03, 1.38) | 1.09 (1.02, 1.16) | Reference | 0.91 (0.80, 1.04) | 0.78 (0.52, 1.15) | 0.053 | 0.363 |
| Cases diagnosed >9 years | 327 | 1.17 (1.02, 1.34) | 1.10 (1.01, 1.19) | Reference | 0.85 (0.72, 1.00) | 0.61 (0.38, 1.00) | 0.072 | 0.649 |
| RPS-IsoP |  |  |  |  |  |  |  |  |
| Cases diagnosed <5 years | 141 | 0.72 (0.41, 1.25) | 0.88 (0.71, 1.09) | Reference | 1.13 (0.93, 1.37) | 1.22 (0.91, 1.63) | 0.197 | 0.263 |
| Cases diagnosed 5-9 years | 136 | 0.91 (0.53, 1.58) | 0.96 (0.77, 1.20) | Reference | 1.04 (0.84, 1.28) | 1.06 (0.77, 1.45) | 0.932 | 0.745 |
| Cases diagnosed >9 years | 327 | 1.42 (0.97, 2.06) | 1.16 (0.99, 1.36) | Reference | 0.84 (0.70, 1.01) | 0.76 (0.56, 1.02) | 0.186 | 0.084 |

Abbreviations: 5-F_2t_-IsoP, 5-F_2t_-Isoprostane; 15-F_2t_-IsoP, 15-F_2t_-Isoprostane; 15-F_2t_-IsoP-M, 2,3-Dinor-5,6-dihydro-15-F_2t_-Isoprostane; RPS-IsoP, ratio of 15-F_2t_-IsoP-M (metabolic product) to 15-F_2t_-IsoP (substrate); CI, confidence interval; ng/mg Cr, nanograms per milligram of creatinine; OR, odds ratio; SMHS, Shanghai Men's Health Study; SWHS, Shanghai Women's Health Study.

^a^ Multivariable ORs estimated using a conditional logistic regression model with restricted cubic-spline functions, stratified by the time interval between the baseline sample collection and cancer diagnosis during follow-up, using the 50th percentile of biomarkers in controls as the reference; and multivariable adjusted for age at baseline, education, cigarette smoking, alcohol consumption, BMI, physical activity, regular use of vitamin supplements, regular use of aspirin and other nonsteroidal anti-inflammatory drugs, Charlson comorbidity score, family history of colorectal cancer in first-degree relatives, and total energy intake.

^b^ Cutoffs were based on the percentile distribution of biomarker levels in controls in the SWHS and SMHS — for 5-F_2t_-IsoP (ng/mg Cr): 1.15 (10th), 1.95 (30th), 2.79 (50th), 4.37 (70th), and 7.90 (90th) in the SWHS and SMHS; for 15-F_2t_-IsoP (ng/mg Cr): 0.64 (10th), 1.00 (30th), 1.20 (50th), 1.91 (70th), and 3.6 (90th); for 15-F_2t_-IsoP-M (ng/mg Cr): 4.30 (10th), 7.27 (30th), 10.70 (50th), 15.88 (70th), and 25.14 (90th); for RPS-IsoP: 272.6% (10th), 563.2% (30th), 810.3% (50th), 1140.9% (70th), and 1950.0% (90th).

^c^ The number of case-control pairs after exclusion of batches with CV > 20%.

**Supplemental Table 12. Associations between baseline urinary F_2_-IsoP levels and subsequent colorectal cancer risk: analyses restricted to batches with CV <20%, without inter-batch correction**

|  | **Number of pairs ^c^** | **OR (95% CI) for colorectal cancer risk relative to reference, ^a^** **by percentile distribution of biomarkers ^b^** | | | | | ***P* for overall association** | ***P* for nonlinear association** |
| --- | --- | --- | --- | --- | --- | --- | --- | --- |
|  |  | **10th** | **30th** | **50th** | **70th** | **90th** |  |  |
| **In the SWHS and SMHS** | | | | | | | | |
| 5-F_2t_-IsoP | 1778 | 1.18 (1.02, 1.38) | 1.08 (1.01, 1.16) | Reference | 0.90 (0.84, 0.96) | 0.79 (0.70, 0.90) | 0.001 | 0.190 |
| 15-F_2t_-IsoP | 1617 | 0.94 (0.83, 1.05) | 0.98 (0.94, 1.02) | Reference | 1.04 (0.96, 1.13) | 1.00 (0.90, 1.12) | 0.174 | 0.182 |
| 15-F_2t_-IsoP-M | 1919 | 1.20 (1.03- 1.39) | 1.10 (1.02- 1.18) | Reference | 0.90 (0.85- 0.95) | 0.78 (0.69- 0.89) | <0.001 | 0.330 |
| RPS-IsoP | 1599 | 1.22 (1.04, 1.43) | 1.09 (1.02, 1.16) | Reference | 0.93 (0.88, 0.99) | 0.90 (0.82, 0.98) | 0.044 | 0.014 |
| **In the SCCS** | | | | | | | | |
| 5-F_2t_-IsoP | 285 | 1.37 (1.06, 1.76) | 1.20 (1.04, 1.39) | Reference | 0.84 (0.73, 0.98) | 0.85 (0.68, 1.06) | 0.030 | 0.010 |
| 15-F_2t_-IsoP | 285 | 1.08 (0.84, 1.41) | 1.03 (0.94, 1.12) | Reference | 0.96 (0.84, 1.09) | 0.91 (0.73, 1.14) | 0.627 | 0.606 |
| 15-F_2t_-IsoP-M | 285 | 1.25 (0.93- 1.67) | 1.10 (0.97- 1.25) | Reference | 0.93 (0.85- 1.03) | 0.95 (0.78- 1.14) | 0.329 | 0.140 |
| RPS-IsoP | 285 | 0.97 (0.69-1.35) | 0.99 (0.87-1.13) | Reference | 0.99 (0.90-1.08) | 0.93 (0.75-1.15) | 0.739 | 0.622 |

Abbreviations: : 5-F_2t_-IsoP, 5-F_2t_-Isoprostane; 15-F_2t_-IsoP, 15-F_2t_-Isoprostane; 15-F_2t_-IsoP-M, 2,3-Dinor-5,6-dihydro-15-F_2t_-Isoprostane; RPS-IsoP, ratio of 15-F_2t_-IsoP-M (metabolic product) to 15-F_2t_-IsoP (substrate); CI, confidence interval; ng/mg Cr, nanograms per milligram of creatinine; OR, odds ratio; SCCS, Southern Community Cohort Study; SMHS, Shanghai Men's Health Study; SWHS, Shanghai Women's Health Study.

^a^ Multivariable ORs estimated using a conditional logistic regression model with restricted cubic-spline functions, using the 50th percentile of biomarkers in controls as the reference; and multivariable adjusted for age at baseline, education, cigarette smoking, alcohol consumption, BMI, physical activity, regular use of vitamin supplements, regular use of aspirin and other nonsteroidal anti-inflammatory drugs, Charlson comorbidity score, family history of colorectal cancer in first-degree relatives, and total energy intake.

^b^ Cutoffs were based on the percentile distribution of biomarker levels in controls—for 5-F_2t_-IsoP (ng/mg Cr): 1.15 (10th), 1.95 (30th), 2.79 (50th), 4.37 (70th), and 7.90 (90th) in the SWHS and SMHS; and 0.74 (10th), 1.18 (30th), 1.95 (50th), 3.08 (70th) and 6.01 (90th) in the SCCS; for 15-F_2t_-IsoP (ng/mg Cr): 0.64 (10th), 1.00 (30th), 1.20 (50th), 1.91 (70th), and 3.6 (90th) in the SWHS and SMHS, and 0.54 (10th), 0.83 (30th), 1.08 (50th), 1.50 (70th) and 2.89 (90th) in the SCCS; for 15-F_2t_-IsoP-M (ng/mg Cr): 4.30 (10th), 7.27 (30th), 10.70 (50th), 15.88 (70th), and 25.14 (90th) in the SWHS and SMHS; and 6.81 (10th), 10.49 (30th), 14.75 (50th), 20.34 (70th) and 32.41 (90th) in the SCCS; for RPS-IsoP: 272.6% (10th), 563.2% (30th), 810.3% (50th), 1140.9% (70th), and 1950.0% (90th) in the SWHS and SMHS, and 553.0% (10th), 1003.3% (30th), 1362.1% (50th), 1835.6% (70th) and 2586.6% (90th) in the SCCS.

^c^ The number of case-control pairs after exclusion of batches with CV >20%, with the case-control ratio of 1:1 in the SWHS, 1:1 in the SMHS, and 1:2 in the SCCS.

**Supplemental Table 13. Associations between baseline urinary F_2_-IsoP levels and subsequent colorectal cancer risk, stratified by follow-up time: analyses restricted to batches with CV <20%, without inter-batch correction**

|  | **Number of pairs ^c^** | **OR (95% CI) for colorectal cancer risk relative to reference, ^a^** **by percentile distribution of biomarkers ^b^** | | | | | ***P* for overall association** | ***P* for nonlinear association** |
| --- | --- | --- | --- | --- | --- | --- | --- | --- |
|  |  | **10th** | **30th** | **50th** | **70th** | **90th** |  |  |
| **Cases diagnosed <5 years after enrollment** | | | | | | | |  |
| 5-F_2t_-IsoP | 387 | 2.01 (1.38- 2.93) | 1.41 (1.17- 1.68) | Reference | 0.63 (0.51- 0.78) | 0.43 (0.30- 0.62) | <0.001 | 0.009 |
| 15-F_2t_-IsoP | 367 | 1.16 (0.94- 1.42) | 1.05 (0.98- 1.13) | Reference | 0.88 (0.74- 1.05) | 0.83 (0.63- 1.10) | 0.388 | 0.180 |
| 15-F_2t_-IsoP-M | 411 | 1.16 (0.86- 1.57) | 1.08 (0.94- 1.24) | Reference | 0.92 (0.81- 1.04) | 0.82 (0.61- 1.09) | 0.352 | 0.654 |
| RPS-IsoP | 349 | 1.04 (0.73- 1.47) | 1.01 (0.88- 1.16) | Reference | 0.99 (0.89- 1.11) | 0.99 (0.84- 1.17) | 0.731 | 0.829 |
| **Cases diagnosed 5-9 years after enrollment** | | | | | | | |  |
| 5-F_2t_-IsoP | 469 | 1.20 (0.92- 1.55) | 1.09 (0.96- 1.24) | Reference | 0.89 (0.77- 1.03) | 0.81 (0.65- 1.01) | 0.169 | 0.284 |
| 15-F_2t_-IsoP | 358 | 0.95 (0.75- 1.21) | 0.98 (0.91- 1.06) | Reference | 1.03 (0.86- 1.24) | 1.00 (0.76- 1.31) | 0.504 | 0.572 |
| 15-F_2t_-IsoP-M | 539 | 1.22 (0.92- 1.62) | 1.11 (0.97- 1.26) | Reference | 0.90 (0.82- 0.99) | 0.80 (0.66- 0.96) | 0.051 | 0.461 |
| RPS-IsoP | 358 | 1.27 (0.90- 1.78) | 1.10 (0.96- 1.27) | Reference | 0.92 (0.82- 1.04) | 0.89 (0.74- 1.06) | 0.382 | 0.171 |
| **Cases diagnosed >9 years after enrollment** | | | | | | | |  |
| 5-F_2t_-IsoP | 922 | 1.04 (0.83- 1.31) | 1.02 (0.93, 1.13) | Reference | 0.96 (0.88, 1.04) | 0.87 (0.70, 1.08) | 0.436 | 0.983 |
| 15-F_2t_-IsoP | 892 | 0.83 (0.69- 1.01) | 0.95 (0.89- 1.00) | Reference | 1.09 (0.98- 1.20) | 1.01 (0.86- 1.19) | 0.123 | 0.045 |
| 15-F_2t_-IsoP-M | 969 | 1.21 (0.96- 1.52) | 1.10 (0.99- 1.24) | Reference | 0.88 (0.81- 0.97) | 0.75 (0.62- 0.91) | 0.011 | 0.575 |
| RPS-IsoP | 892 | 1.31 (1.05- 1.62) | 1.12 (1.02- 1.24) | Reference | 0.89 (0.81- 0.98) | 0.84 (0.72- 0.98) | 0.046 | 0.013 |

Abbreviations: : 5-F_2t_-IsoP, 5-F_2t_-Isoprostane; 15-F_2t_-IsoP, 15-F_2t_-Isoprostane; 15-F_2t_-IsoP-M, 2,3-Dinor-5,6-dihydro-15-F_2t_-Isoprostane; RPS-IsoP, ratio of 15-F_2t_-IsoP-M (metabolic product) to 15-F_2t_-IsoP (substrate); CI, confidence interval; ng/mg Cr, nanograms per milligram of creatinine; OR, odds ratio; SMHS, Shanghai Men's Health Study; SWHS, Shanghai Women's Health Study.

^a^ Multivariable ORs estimated using a conditional logistic regression model with restricted cubic-spline functions, stratified by the time interval between the baseline sample collection and cancer diagnosis during follow-up, using the 50th percentile of biomarkers in controls as the reference; and multivariable adjusted for age at baseline, education, cigarette smoking, alcohol consumption, BMI, physical activity, regular use of vitamin supplements, regular use of aspirin and other nonsteroidal anti-inflammatory drugs, Charlson comorbidity score, family history of colorectal cancer in first-degree relatives, and total energy intake.

^b^ Cutoffs were based on the percentile distribution of biomarker levels in controls in the SWHS and SMHS — for 5-F_2t_-IsoP (ng/mg Cr): 1.15 (10th), 1.95 (30th), 2.79 (50th), 4.37 (70th), and 7.90 (90th); for 15-F_2t_-IsoP (ng/mg Cr): 0.64 (10th), 1.00 (30th), 1.20 (50th), 1.91 (70th), and 3.6 (90th); for 15-F_2t_-IsoP-M (ng/mg Cr): 4.30 (10th), 7.27 (30th), 10.70 (50th), 15.88 (70th), and 25.14 (90th); for RPS-IsoP: 272.6% (10th), 563.2% (30th), 810.3% (50th), 1140.9% (70th), and 1950.0% (90th).

^c^ The number of case-control pairs after exclusion of batches with CV >20%.

**Supplemental Table 14. Associations between baseline urinary F_2_-IsoP levels and subsequent colorectal cancer risk: analyses including all participants without inter-batch correction**

|  | **OR (95% CI) for colorectal cancer risk relative to reference, ^a^** **by percentile distribution of biomarkers ^b^** | | | | | ***P* for overall association** | ***P* for nonlinear association** | |
| --- | --- | --- | --- | --- | --- | --- | --- | --- |
|  | **10th** | **30th** | **50th** | **70th** | **90th** |  |  | |
| **In the SWHS and SMHS** | | | | | | | |  |
| 5-F_2t_-IsoP | 1.19 (1.04, 1.36) | 1.09 (1.02, 1.16) | Reference | 0.90 (0.84, 0.96) | 0.80 (0.71, 0.90) | 0.001 | 0.108 | |
| 15-F_2t_-IsoP | 1.00 (0.91, 1.10) | 1.00 (0.97, 1.03) | Reference | 0.99 (0.93, 1.06) | 0.95 (0.86, 1.05) | 0.194 | 0.758 | |
| 15-F_2t_-IsoP-M | 1.25 (1.06, 1.47) | 1.12 (1.03, 1.21) | Reference | 0.90 (0.85, 0.95) | 0.79 (0.70, 0.89) | 0.000 | 0.197 | |
| RPS-IsoP | 1.15 (1.02, 1.30) | 1.07 (1.01, 1.13) | Reference | 0.95 (0.90, 0.99) | 0.92 (0.85, 0.99) | 0.060 | 0.022 | |
| **In the SCCS** | | | | | | | |  |
| 5-F_2t_-IsoP | 1.37 (1.06, 1.76) | 1.20 (1.04, 1.39) | Reference | 0.84 (0.73, 0.98) | 0.85 (0.68, 1.06) | 0.03 | 0.01 | |
| 15-F_2t_-IsoP | 1.08 (0.84, 1.41) | 1.03 (0.94, 1.12) | Reference | 0.96 (0.84, 1.09) | 0.91 (0.73, 1.14) | 0.627 | 0.606 | |
| 15-F_2t_-IsoP-M | 1.25 (0.93, 1.67) | 1.10 (0.97, 1.25) | Reference | 0.93 (0.85, 1.03) | 0.95 (0.78, 1.14) | 0.329 | 0.14 | |
| RPS-IsoP | 0.97 (0.69, 1.35) | 0.99 (0.87, 1.13) | Reference | 0.99 (0.90, 1.08) | 0.93 (0.75, 1.15) | 0.739 | 0.622 | |

Abbreviations: 5-F_2t_-IsoP, 5-F_2t_-Isoprostane; 15-F_2t_-IsoP, 15-F_2t_-Isoprostane; 15-F_2t_-IsoP-M, 2,3-Dinor-5,6-dihydro-15-F_2t_-Isoprostane; RPS-IsoP, ratio of 15-F2t-IsoP-M (metabolic product) to 15-F2t-IsoP (substrate); CI, confidence interval; ng/mg Cr, nanograms per milligram of creatinine; OR, odds ratio; SCCS, Southern Community Cohort Study; SMHS, Shanghai Men's Health Study; SWHS, Shanghai Women's Health Study.

^a^ The OR was estimated using a conditional logistic regression model with restricted cubic-spline functions, with the 50th percentile of biomarkers in controls treated as the reference, multivariable adjusted for age at baseline, education, cigarette smoking, alcohol consumption, BMI, physical activity, regular use of vitamin supplements, regular use of aspirin and other nonsteroidal anti-inflammatory drugs, Charlson comorbidity score, family history of colorectal cancer in first-degree relatives, and total energy intake. The number of case-control pairs included in the analysis without batch-CV-based exclusion was 1938 in the SWHS and SMHS and 285 in the SCCS, with the case-control ratio of 1:1 in the SWHS and SMHS, and 1:2 in the SCCS.

^b^ Cutoffs were based on the percentile distribution of biomarker levels in controls—for 5-F_2t_-IsoP (ng/mg Cr): 1.15 (10th), 1.95 (30th), 2.79 (50th), 4.37 (70th), and 7.90 (90th) in the SWHS and SMHS; and 0.74 (10th), 1.18 (30th), 1.95 (50th), 3.08 (70th) and 6.01 (90th) in the SCCS; for 15-F_2t_-IsoP (ng/mg Cr): 0.64 (10th), 1.00 (30th), 1.20 (50th), 1.91 (70th), and 3.6 (90th) in the SWHS and SMHS, and 0.54 (10th), 0.83 (30th), 1.08 (50th), 1.50 (70th) and 2.89 (90th) in the SCCS. for 15-F_2t_-IsoP-M (ng/mg Cr): 4.30 (10th), 7.27 (30th), 10.70 (50th), 15.88 (70th), and 25.14 (90th) in the SWHS and SMHS; and 6.81 (10th), 10.49 (30th), 14.75 (50th), 20.34 (70th) and 32.41 (90th) in the SCCS; for RPS-IsoP: 272.6% (10th), 563.2% (30th), 810.3% (50th), 1140.9% (70th), and 1950.0% (90th) in the SWHS and SMHS, and 553.0% (10th), 1003.3% (30th), 1362.1% (50th), 1835.6% (70th) and 2586.6% (90th) in the SCCS.

**Supplemental Table 15. Associations between baseline urinary F_2_-IsoP levels and subsequent colorectal cancer risk, stratified by follow-up time: analyses including all participants from the SWHS and SMHS, without inter-batch correction**

|  | **OR (95% CI) for colorectal cancer risk relative to reference,^s^** **by percentile distribution of biomarkers ^b^** | | | | | ***P* for overall association** | | | ***P* for nonlinear association** | | |
| --- | --- | --- | --- | --- | --- | --- | --- | --- | --- | --- | --- |
|  | **10th** | **30th** | **50th** | **70th** | **90th** |  | |  | | |  |
| **Cases diagnosed <5 years after enrollment** | | | | | | |  | | |  |  |
| 5-F_2t_-IsoP | 2.09 (1.39, 3.13) | 1.52 (1.21, 1.90) | Reference | 0.74 (0.65, 0.85) | 0.62 (0.46, 0.83) | 0.000 | | 0.011 | | |  |
| 15-F_2t_-IsoP | 1.21 (0.94, 1.55) | 1.08 (0.98, 1.19) | Reference | 0.91 (0.80, 1.03) | 0.89 (0.73, 1.08) | 0.335 | | 0.152 | | |  |
| 15-F_2t_-IsoP-M | 1.43 (0.99, 2.06) | 1.20 (1.00, 1.46) | Reference | 0.85 (0.73, 0.99) | 0.76 (0.55, 1.05) | 0.116 | | 0.140 | | |  |
| RPS-IsoP | 1.02 (0.81, 1.28) | 1.01 (0.92, 1.11) | Reference | 0.99 (0.88, 1.12) | 0.98 (0.71, 1.35) | 0.969 | | 0.879 | | |  |
| **Cases diagnosed 5-9 years after enrollment** | | | | | | |  | | |  |  |
| 5-F_2t_-IsoP | 1.32 (1.00, 1.75) | 1.16 (1.00, 1.34) | Reference | 0.87 (0.76, 0.98) | 0.79 (0.66, 0.96) | 0.050 | | 0.116 | | |  |
| 15-F_2t_-IsoP | 1.01 (0.83, 1.22) | 1.00 (0.92, 1.10) | Reference | 0.98 (0.85, 1.12) | 0.89 (0.73, 1.08) | 0.230 | | 0.806 | | |  |
| 15-F_2t_-IsoP-M | 1.25 (0.90, 1.75) | 1.11 (0.96, 1.28) | Reference | 0.91 (0.84, 1.00) | 0.81 (0.67, 0.96) | 0.053 | | 0.464 | | |  |
| RPS-IsoP | 1.09 (0.88, 1.33) | 1.03 (0.95, 1.13) | Reference | 0.96 (0.87, 1.06) | 0.95 (0.82, 1.09) | 0.680 | | 0.433 | | |  |
| **Cases diagnosed >9 years after enrollment** | | | | | | |  | | |  |  |
| 5-F_2t_-IsoP | 1.01 (0.83, 1.23) | 1.01 (0.92, 1.10) | Reference | 0.99 (0.91, 1.06) | 0.91 (0.77, 1.07) | 0.439 | | 0.789 | | |  |
| 15-F_2t_-IsoP | 0.92 (0.79, 1.06) | 0.98 (0.95, 1.01) | Reference | 1.05 (0.96, 1.16) | 1.04 (0.90, 1.20) | 0.361 | | 0.201 | | |  |
| 15-F_2t_-IsoP-M | 1.20 (0.94, 1.53) | 1.10 (0.98, 1.25) | Reference | 0.90 (0.83, 0.97) | 0.77 (0.65, 0.93) | 0.012 | | 0.752 | | |  |
| RPS-IsoP | 1.29 (1.08, 1.55) | 1.12 (1.03, 1.22) | Reference | 0.93 (0.88, 0.98) | 0.90 (0.83, 0.98) | 0.020 | | 0.006 | | |  |

Abbreviations: 5-F_2t_-IsoP, 5-F_2t_-Isoprostane; 15-F_2t_-IsoP, 15-F_2t_-Isoprostane; 15-F_2t_-IsoP-M, 2,3-Dinor-5,6-dihydro-15-F_2t_-Isoprostane; RPS-IsoP, ratio of 15-F_2t_-IsoP-M (β-oxidation product) to 15-F_2t_-IsoP (substrate); CI, confidence interval; ng/mg Cr, nanograms per milligram of creatinine; OR, odds ratio; SMHS, Shanghai Men's Health Study; SWHS, Shanghai Women's Health Study.

^a^ The OR was estimated using a conditional logistic regression model with restricted cubic-spline functions, with the 50th percentile of biomarkers in controls treated as the reference, multivariable adjusted for age at baseline, education, cigarette smoking, alcohol consumption, BMI, physical activity, regular use of vitamin supplements, regular use of aspirin and other nonsteroidal anti-inflammatory drugs, Charlson comorbidity score, family history of colorectal cancer in first-degree relatives, and total energy intake. The number of case-control pairs without batch-CV-based exclusion in SWHS and SMHS was 429 pairs in the cases diagnosed <5 years, 540 pairs in the cases diagnosed 5-9 years, and 969 pairs in the cases diagnosed >9 years, with the case-control ratio of 1:1.

^b^ Cutoffs were based on the percentile distribution of biomarker levels in controls in the SWHS and SMHS — for 5-F_2t_-IsoP (ng/mg Cr): 1.15 (10th), 1.95 (30th), 2.79 (50th), 4.37 (70th), and 7.90 (90th); for 15-F_2t_-IsoP (ng/mg Cr): 0.64 (10th), 1.00 (30th), 1.20 (50th), 1.91 (70th), and 3.6 (90th); for 15-F_2t_-IsoP-M (ng/mg Cr): 4.30 (10th), 7.27 (30th), 10.70 (50th), 15.88 (70th), and 25.14 (90th); for RPS-IsoP: 272.6% (10th), 563.2% (30th), 810.3% (50th), 1140.9% (70th), and 1950.0% (90th).
**Supplemental Table 16. Associations between a composite index of oxidative stress markers and subsequent risk of colorectal cancer**

|  | **Number of pairs ^c^** | **OR (95% CI) for colorectal cancer risk relative to reference, ^a^** **by percentile distribution of the index ^b^** | | | | | ***P* for overall association** | ***P* for nonlinear association** |
| --- | --- | --- | --- | --- | --- | --- | --- | --- |
|  |  | **10th** | **30th** | **50th** | **70th** | **90th** |  |  |
| **In the SWHS and SMHS** | 1778 | 1.28 (1.09, 1.51) | 1.12 (1.04, 1.20) | Reference | 0.88 (0.83, 0.94) | 0.77 (0.67, 0.87) | <0.001 | 0.088 |
| **In the SCCS** | 285 | 1.69 (1.24, 2.30) | 1.21 (1.08, 1.35) | Reference | 0.86 (0.78, 0.95) | 0.89 (0.75, 1.06) | 0.004 | 0.001 |

Abbreviations: 5-F_2t_-IsoP, 5-F_2t_-Isoprostane; 8-oxo-dG, 8-oxo-7,8-dihydro-2'-deoxyguanosine; 8-oxo-Guo, 7,8-dihydro-8-oxo-guanosine; CI, confidence interval; ng/mg Cr, nanograms per milligram of creatinine; OR, odds ratio; SCCS, Southern Community Cohort Study; SMHS, Shanghai Men's Health Study; SWHS, Shanghai Women's Health Study.

^a^ The OR was estimated by using a conditional logistic regression model with restricted cubic-spline functions, with the 50th percentile of the index in controls treated as the reference, and multivariable adjusted for age at baseline, education, cigarette smoking, alcohol consumption, BMI, physical activity, regular use of vitamin supplements, regular use of aspirin and other nonsteroidal anti-inflammatory drugs, Charlson comorbidity score, family history of colorectal cancer in first-degree relatives, and total energy intake.

^b^ The index is derived from the first principal component (PC1) from a principal component analysis (PCA) of three OxS markers: 5-F_2t_-IsoP, 8-oxo-dG and 8-oxo-Guo. Cutoffs were based on the percentile distribution of the index levels in controls— -1.03 (10th), -0.67 (30th), -0.26 (50th), 0.290 (70th), and 1.54 (90th) in the SWHS and the SMHS; and –1.03 (10th), -0.69 (30th), -0.27 (50th), 0.22 (70th) and 1.60 (90th) in the SCCS.

^c^ The number of case-control pairs after exclusion of batches with CV >20%, with case-control ratio of 1:1 in the SWHS, 1:1 in the SMHS and 1:2 in the SCCS.

**Supplemental Figure 1. Participant Flow Chart**

**Shanghai Women’s Health Study, SWHS** (n=74,940)

**Shanghai Men’s Health Study, SWMS** (n=61,469)

**Southern Community Cohort Study, SCCS** (n=84,507)

**Exclusion**

Invalid baseline data (n=1023)

Baseline urine sample unavailable (n=60,598)

Loss of follow-up for CRC (n=0)

**Exclusion**

Prevalent cancer (n=1598)

Baseline urine sample unavailable (=15,898)

Loss of follow-up (n=409)

**Exclusion**

Prevalent cancer (n=1703)

**Participants included for the replication analysis**

Incident patients with colorectal cancer as the first primary cancer (n=285)

1:2 individually matched controls (n=570)

**Participants included for the primary analysis**

Incident patients with colorectal cancer as the first primary cancer (n=1938)

1:1 individually matched controls (n=1938)

**Cancer-free participants with baseline urine samples** (n=21,183), median 13 years of follow-up

**Participants with baseline urine samples** (n=22,886)

**Cancer-free participants with baseline urine samples** (n=118,905), median 15 years of follow-up

**Supplemental Figure 2. Distribution of follow‑up time from enrollment to CRC diagnosis**

**Supplemental Figure 3. Correlations among oxidative stress markers of nucleic acids and lipids in urine**

**Supplemental Figure 4**

**Supplemental Figure 4. Smoothed plot of multivariable-adjusted ORs for colorectal cancer risk according to urinary levels of 15-F_2t_-IsoP, by time interval from baseline to cancer diagnosis: Overall (A), <5 years (B), 5-9 years (C), and >9 years (D) in the SWHS and the SMHS.**

The OR was estimated using a conditional logistic regression model with restricted cubic spline functions, with the 50th percentile of biomarker levels in controls as the reference and adjusted for potential confounding factors as listed in the footnotes of Table 1. OR values on the *y* axis are shown in log scale. The solid line indicates the point estimate, and the dashed lines indicate the 95% CI. *P* for heterogeneity across time interval groups was 0.361.

Abbreviations: 15-F_2t_-IsoP, 15-F_2t_-Isoprostane; CI, confidence interval; ng/mg Cr, nanograms per milligram of creatinine; OR, odds ratio; SMHS, Shanghai Men's Health Study; SWHS, Shanghai Women's Health Study.

**Supplemental Figure 5**

**Supplemental Figure 5. Smoothed plot of multivariable-adjusted ORs for colorectal cancer risk according to urinary levels of 15-F_2t_-IsoP-M, by time interval from baseline to cancer diagnosis: Overall (A), <5 years (B), 5-9 years (C), and >9 years (D) in the SWHS and the SMHS.**

The OR was estimated using a conditional logistic regression model with restricted cubic spline functions, with the 50th percentile of biomarker levels in controls as the reference and adjusted for potential confounding factors as listed in the footnotes of Table 1. OR values on the *y* axis are shown in log scale. The solid line indicates the point estimate, and the dashed lines indicate the 95% CI. *P* for heterogeneity across time interval groups was 0.734.

Abbreviations: 15-F_2t_-IsoP-M, 2,3-Dinor-5,6-dihydro-15-F_2t_-Isoprostane; CI, confidence interval; ng/mg Cr, nanograms per milligram of creatinine; OR, odds ratio; SMHS, Shanghai Men's Health Study; SWHS, Shanghai Women's Health Study.

**Supplemental Figure 6**

**Supplemental Figure 6. Smoothed plot of multivariable-adjusted ORs for colorectal cancer risk according to RPS-IsoP, by time interval from baseline to cancer diagnosis: Overall (A), <5 years (B), 5-9 years (C), and >9 years (D) in the SWHS and the SMHS.**

The OR was estimated using a conditional logistic regression model with restricted cubic spline functions, with the 50th percentile of biomarker levels in controls as the reference and adjusted for potential confounding factors as listed in the footnotes of Table 1. OR values on the *y* axis are shown in log scale. The solid line indicates the point estimate, and the dashed lines indicate the 95% CI. *P* for heterogeneity across time interval groups was 0.095.

Abbreviations: RPS-IsoP, ratio of 15-F_2t_-IsoP-M (β-oxidation product) to 15-F_2t_-IsoP (substrate); CI, confidence interval; ng/mg Cr, nanograms per milligram of creatinine; OR, odds ratio; SMHS, Shanghai Men's Health Study; SWHS, Shanghai Women's Health Study.
